# Evaluation and optimization of the syndromic management of female genital tract infections in Nairobi, Kenya

**DOI:** 10.1186/s12879-023-08442-2

**Published:** 2023-08-22

**Authors:** Gloria S. Omosa-Manyonyi, Marloes de Kam, Alma Tostmann, Mwasi A. Masido, Nyawira Nyagah, Moses M. Obimbo, Andre J.A.M. van der Ven, Jaap ten Oever

**Affiliations:** 1 Faculty of Health Sciences, University of Nairobi, Nairobi, Kenya; 2 Department of Internal Medicine, Radboud Center for Infectious Diseases, Radboud University Medical Center, Nijmegen, NL Netherlands; 3 Department of Medical Microbiology, Radboud Center for Infectious Diseases, Radboud University Medical Center, Nijmegen, NL Netherlands; 4 Nairobi City County, Health Services, Nairobi, Kenya

**Keywords:** Lower genital tract symptoms, Vaginal discharge, Syndromic treatment, Vaginitis, Genital tract infections, Performance, Kenya

## Abstract

**Background:**

Genital tract infections pose a public health concern. In many low-middle-income countries, symptom-based algorithms guide treatment decisions. Advantages notwithstanding, this strategy has important limitations. We aimed to determine the infections causing lower genital tract symptoms in women, evaluated the Kenyan syndromic treatment algorithm for vaginal discharge, and proposed an improved algorithm.

**Methods:**

This cross-sectional study included symptomatic non-pregnant adult women presenting with lower genital tract symptoms at seven outpatient health facilities in Nairobi. Clinical, socio-demographic information and vaginal swabs microbiological tests were obtained. Multivariate logistic regression analyses were performed to find predictive factors for the genital infections and used to develop an alternative vaginal discharge treatment algorithm (using 60% of the dataset). The other 40% of data was used to assess the performance of each algorithm compared to laboratory diagnosis.

**Results:**

Of 813 women, 66% had an infection (vulvovaginal candidiasis 40%, bacterial vaginosis 17%, *Neisseria gonorrhoea* 14%, multiple infections 23%); 56% of women reported ≥ 3 lower genital tract symptoms episodes in the preceding 12 months. Vulvovaginal itch predicted vulvovaginal candidiasis (odds ratio (OR) 2.20, 95% CI 1.40–3.46); foul-smelling vaginal discharge predicted bacterial vaginosis (OR 3.63, 95% CI 2.17–6.07), and sexually transmitted infection (*Neisseria gonorrhoea, Trichomonas vaginalis*, *Chlamydia trachomatis*, *Mycoplasma genitalium*) (OR 1.64, 95% CI 1.06–2.55). Additionally, lower abdominal pain (OR 1.73, 95% CI 1.07–2.79) predicted sexually transmitted infection. Inappropriate treatment was 117% and 75% by the current and alternative algorithms respectively. Treatment specificity for bacterial vaginosis/*Trichomonas vaginalis* was 27% and 82% by the current and alternative algorithms, respectively. Performance by other parameters was poor to moderate and comparable between the two algorithms.

**Conclusion:**

Single and multiple genital infections are common among women presenting with lower genital tract symptoms at outpatient clinics in Nairobi. The conventional vaginal discharge treatment algorithm performed poorly, while the alternative algorithm achieved only modest improvement. For optimal care of vaginal discharge syndrome, we recommend the inclusion of point-of-care diagnostics in the flowcharts.

**Supplementary Information:**

The online version contains supplementary material available at 10.1186/s12879-023-08442-2.

## Introduction

Female genital tract infections constitute a significant public health problem with high disease burden [1–3], which is even higher among pregnant women and those living with HIV [4, 5]. Common symptoms associated with lower genital tract infections (LGTI) include vaginal discharge, dysuria, lower abdominal pain, dyspareunia, and pruritus. Vaginal discharge is commonest among these, being present in up to 75% of women with LGTI [6, 7]. However, these symptoms are not specific for LGTI, for example, in sub-Saharan African studies 27–49% of women with vaginal discharge did not have an infection [8, 9]. Presence of vaginal discharge does not necessarily imply a manifestation of a pathological condition; it could be present as a normal physiological phenomenon.

The commonly detected LGTI include vulvovaginal candidiasis (VVC), bacterial vaginosis (BV) and sexually transmitted infections (STI) caused by *Trichomonas vaginalis* (TV), *Neisseria gonorrhoea* (NG), *Chlamydia trachomatis* (CT), and *Mycoplasma genitalium* (MG) [9]. The infections are responsible for significant morbidity in women negatively affecting quality of life, increasing the risk of HIV transmission, and possibly causing adverse gynaecological and obstetrical outcomes [10–12].

**Fig. 1:**
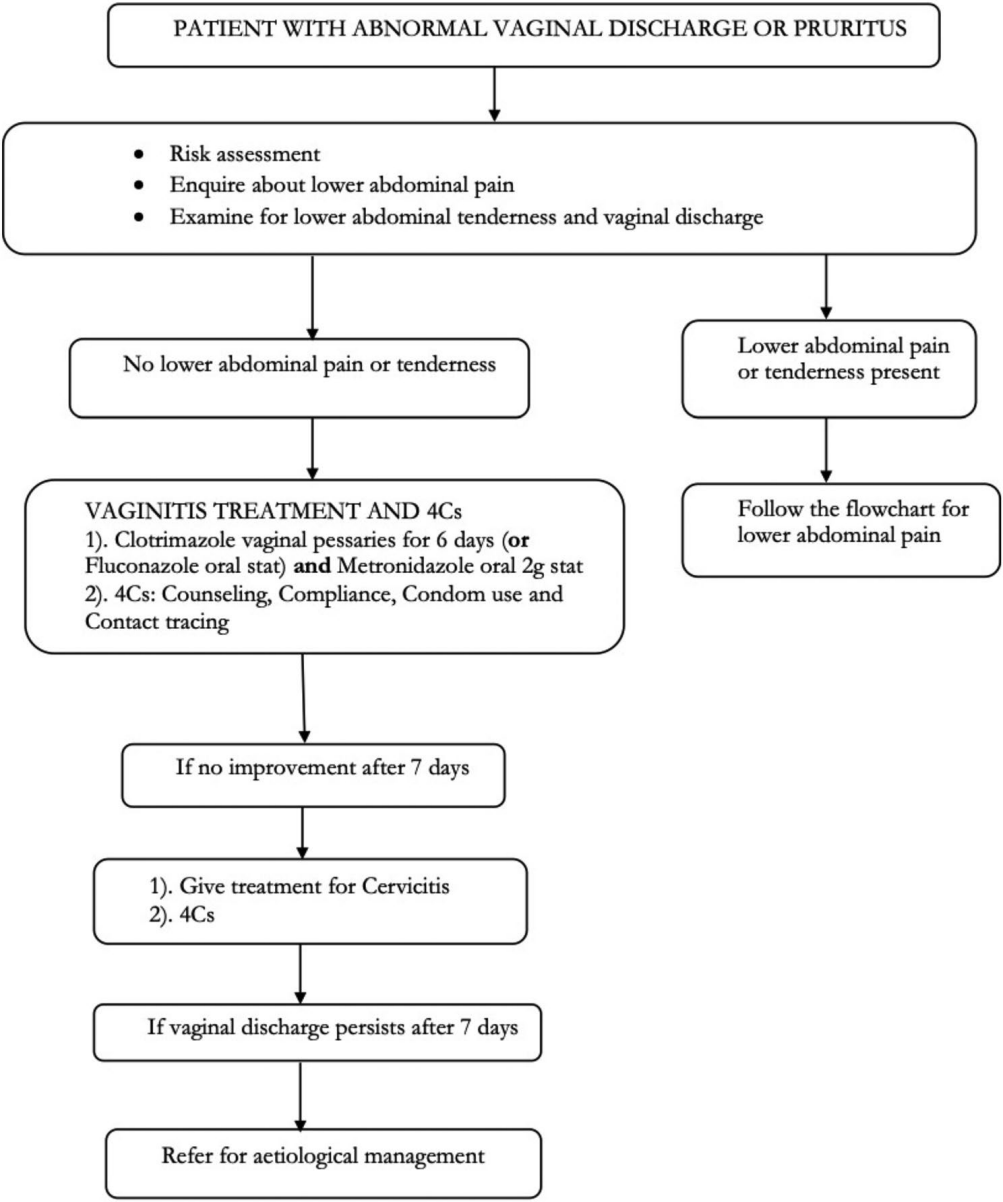
Algorithm used in Kenya for management of Vaginal Discharge Syndrome [13] Legend: A risk assessment is performed, enquiry made on lower abdominal pain, and examination for lower abdominal tenderness carried out. Patients without lower abdominal pain or lower abdominal tenderness are given treatment for vaginitis which includes antifungal medication for VVC and metronidazole for BV and TV. Those with lower abdominal pain or lower abdominal tenderness are managed using the lower abdominal pain (LAP) flowchart. Patients with unresolved symptoms after 7 days are given treatment for cervicitis. Laboratory testing is recommended on the 3rd visit, fourteen days later, for those with persistent vaginal discharge

In many low- and middle-income countries, syndromic algorithms based on genital tract symptoms guide treatment decisions for genital tract infections. The Kenyan guidelines for reproductive tract infections 2018 [13] recommends a syndromic approach using algorithms based on the World Health Organization (WHO) guidelines [14]. For patients presenting with vaginal discharge and vulvovaginal itch, the algorithm for management of vaginal discharge syndrome is applied [13] (Fig. 1).

The use of laboratory tests to diagnose LGTI allows species identification and tailored treatment. Yet, these tests are not widely applied in many resource-limited countries, but are reserved for women whose symptoms persist after the syndromic treatment. The syndromic approach has the advantage of providing treatment to patients immediately at the initial visit and without laboratory-associated delays and costs [15]. Additionally, rapid initiation of treatment reduces further transmission of infections and increases treatment coverage. However, the downside of this approach is under-treatment or delayed treatment of infections due to misdiagnosis or a missed diagnosis, as well as overtreatment and therefore the unnecessary use of antimicrobials contributing to the development of antimicrobial resistance [15].

Studies have shown that the syndromic approach is inadequate in the management of STI [16–19]; however, there is scarcity of data from Kenya interrogating the performance of the syndromic approach in the management of vaginal discharge syndrome/vaginitis. Hence, the question is whether the present guideline for management of vaginitis is performing well and if there is a need to improve it. The present study therefore aimed to evaluate and improve the performance of the vaginal discharge syndromic treatment algorithm currently in use in Kenya for the management of women presenting with lower genital tract symptoms (LGTS). For this purpose, we (1) analysed the LGTI causing the LGTS; (2) determined the social, demographic, behavioural, and clinical characteristics associated with the LGTI; (3) assessed the performance of the currently used vaginal discharge syndrome algorithm; (4) developed and evaluated an alternative algorithm.

## Methods

### Study aim, design and setting

This was a cross-sectional study to evaluate and improve the performance of the vaginal discharge syndromic treatment algorithm currently used in Kenya. The study was part of a larger study on VVC and recurrent VVC (RVVC), conducted between October 2018 and March 2020, among adult women presenting with LGTS at seven outpatient health facilities in Nairobi City County (NCC), Kenya. These health facilities serve a non-exclusive/ordinary population, and attend to large volumes of patients. During the study period, the management of LGTS followed a syndromic approach as described in the Kenyan guidelines for reproductive tract infections 2018 [13].

### Inclusion and exclusion criteria

Included were women aged 18–50 years, presenting with LGTS, who gave informed consent. Participants were excluded if they were pregnant, menopausal, had genital malignancy, or tested positive for HIV or glycosuria, as determined in the larger study on VVC and RVVC. Diabetes mellitus, HIV and pregnancy are established factors associated with increased risk for various LGTI; to be more representative of the general population, we therefore excluded women with these conditions.

### Participant recruitment and data collection

Health care workers at the participating outpatient clinics identified patients with LGTS during medical history taking and informed them of the study; interested patients were referred to the research room within the health facility prior to physical examination. At the research room, the study nurse gave study information to potential participants using the informed consent document, after which individuals willing to join the study provided informed consent. The following information was then collected using a standardized questionnaire: socio-demographic data (age, marital status, education, occupation, and ethnicity/tribe), sexual behaviour, vaginal practices (douching, use of inserts), medical history including the occurrence of previous episodes of LGTS, and use of medications. Thereafter a urine sample was obtained for pregnancy and dipstick testing; pregnant participants were excluded from further participation and referred to the health facility’s antenatal clinic. Measurements of temperature, weight, height, pulse rate, and blood pressure were obtained. A clinical officer then conducted a physical examination directed by symptoms, including vulvovaginal examination, and obtained vaginal swabs specimens via a vaginal speculum. Subsequently, the clinical officer offered treatment using the syndromic treatment guidelines (Fig. 1) [13]. Next, HIV counselling and testing was done and the results released in real time to the participant. Participants were asked to make a study follow-up visit for possible adaptation of the given treatment, based on the laboratory test results.

### Laboratory testing

Urine was used to test for pregnancy by detection of Human Chorionic Gonadotropin, and for glycosuria using dipstick test. HIV-1 counselling and testing was performed according to the Kenya National HIV testing guideline, at the clinic in real-time with rapid-kit-testing using blood from finger pricking [20]. Vaginal smear specimens were tested for candidiasis by microscopic examination and culture on Sabouraud dextrose agar media; BV by the Nugent score; and for CT, NG, MG, and TV by multiplex Real Time polymerase chain reaction (PCR) test (Sacace Biotechnologies, Como Italy).

### Outcomes measures

The outcome measures were: (1) the aetiology of LGTS (2) the association between patient characteristics (social, demographic, behavioural and clinical) and the aetiology of LGTS (3) the performance of the current vaginal discharge syndrome algorithm with respect to recommending appropriate/correct treatment for the LGTI (4) the development and performance of an alternative algorithm incorporating logistic regression-derived variables from outcome 2.

### Definitions

Vaginal discharge with or without presence of foul smell, vulvar or vaginal itch/pruritus, vulvovaginal soreness or burning sensation, lower abdominal pain, dysuria, and dyspareunia were all defined as LGTS.

We distinguished VVC, BV, TV, CT, NG, and MG as cause of LGTI. STI were the infections caused by TV, CT, NG, and MG. VVC was defined as at least one positive test from either direct microscopic examination or culture on Sabouraud dextrose agar. BV was defined as a Nugent score of 7 or above. CT, NG, MG, and TV were diagnosed by a positive PCR test. If all microbiological tests were negative, the LGTS were regarded to have a non-infectious cause.

Recurrence of LGTS was defined as 3 or more episodes of LGTS in the preceding 12 months, including the episode at the study visit; in women with confirmed VVC, this was defined as RVVC.

We defined the performance of a treatment algorithm (current or alternative algorithm) as the ability of the algorithm to recommend appropriate treatment according to the laboratory-based diagnosis.

For evaluation of the algorithms’ performance, we classified the algorithm-based treatment recommendations as correct, inappropriate or missed. A treatment was defined as correct when the algorithm-recommended treatment was consistent with the microbiological diagnosis. For BV and TV, receiving metronidazole when either one was present was considered correct; for VVC the correct treatment was vaginal or oral antifungal. Treatment was regarded as inappropriate if a patient received irrelevant treatment with reference to the laboratory test results. There was missed treatment if the correct treatment would not be recommended despite a laboratory-confirmed infection. We defined correct treatment for any of CT, NG, and MG as referral to the lower abdominal pain (LAP) flowchart; by this we classified all VVC or BV-TV referred to the LAP by the current flowchart as missed treatment.

A patient could be classified into the correct treatment, and/or inappropriate treatment, and/or missed treatment categories at the same time.

### Data analysis

Descriptive analyses consisted of frequencies (including percentages), mean (including standard deviation), and/or ranges as appropriate, to describe the study population and potential predictors. As an initial step to relate independent variables to the aetiology of LTGS, we calculated odds ratios (OR) and determined statistical significance using Chi-Square statistics. To further assess possible predictors of an aetiology/infection, multivariate binary logistic regression analyses were performed. We started with the purest groups, the mono-infections; patients with multiple infections were therefore included in more than one regression analysis.

The data were then randomly divided into a training and validation dataset in a ratio of 60:40 respectively [21]. A backward conditional logistic multivariate regression method was used on the training (60%) dataset. All statistically significant variables from the bivariate analysis were included in the model. With the most discriminative and plausible variables from this multivariate analysis an alternative algorithm was developed, while considering the relative frequency of each infection and predictor. The remaining 40% of the data were used to assess the algorithms’ performance. For each patient in the validation dataset, correct, inappropriate, and/or missed treatment were assessed as defined above, and summarized for each treatment option in the current or alternative algorithm. In the current algorithm, treatment options were for VVC and BV-TV, and LAP referral. The alternative algorithm had the treatment options for VVC, BV-TV, LAP referral, and no infection. A McNemar test was performed to compare the classification of treatment groups, between the current and alternative algorithms.

The algorithm performance results obtained from the mono-infections did not warrant us to proceed to the next step i.e. to include the most common combinations of infections in the regression analysis. Hence the potential association between different types of infections was not assessed in this study.

We further determined the sensitivity, specificity, positive predictive value (PPV), and negative predictive value (NPV) of the current and alternative algorithms for treatment allocation. Calculations were made using the laboratory test results as a reference or ‘gold standard’. Sensitivity represented the probability that a patient with a certain infection or syndrome was correctly assigned by the algorithm to a treatment of that infection or syndrome. Specificity represented the percentage of patients without an infection who were assigned as not having to receive treatment by the algorithm. PPV was the probability that a patient who was allocated to an infection- or syndrome-specific treatment did in fact have that infection or syndrome; NPV as the probability that a patient who was not allocated to an infection-specific or syndrome-specific treatment would indeed not have that condition.

All analyses were performed using IBM SPSS Statistical Software (version 25). A p-value of < 0.05 was considered statistically significant.

## Results

### Participants’ characteristics

Of the 856 women who provided written informed consent for the parent study, 8 (1%) were excluded due incomplete data, and 35 (4%) due to pregnancy (n = 24) and HIV positivity (n = 11), leaving a total of 813 participants for this analysis. The mean age of the participants was 29.5 years (standard deviation 7.1, range 18 to 50 years); 55% were married, and 34% were housewives or unemployed. Contraceptive (excluding condoms) use in the preceding 12 months was in 65%, and condom use during the preceding three months in 26% of participants, and 85% reported only 1 sexual partner in the preceding 3 months. (Table 1).

**Table 1:** Socio-demographic, clinical and sexual-behavioural characteristics of women presenting with LGTS at NCC outpatient clinics, Kenya

Characteristic		Total n (%)^#^	Infection n (%)	No infection n (%)	Odds Ratio^¥^ (95% CI)	p-value
**Overall**		813 (100)	540 (66.4)	273 (33.6)		
**Age**	18–25 years	306 (38.4)	214 (69.9)	92 (30.1)	ref*	
	26–35 years	325 (40.8)	211 (64.9)	114 (35.1)	0.80 (0.57–1.11)	0.18
	> 35 years	166 (20.8)	105 (63.3)	61 (36.7)	0.74 (0.50–1.10)	0.14
**Marital status**	Single	271 (33.4)	186 (68.6)	85 (31.4)	ref*	
	Married	445 (54.8)	290 (65.2)	155 (34.8)	0.86 (0.62–1.18)	0.34
	Separated/divorced/widow	96 (11.8)	64 (66.7)	32 (33.3)	0.91 (0.56–1.50)	0.72
**Occupation**	Unemployed, housewife	279 (34.4)	183 (65.6)	96 (34.4)	ref*	
	Professional worker	101 (12.4)	76 (75.2)	25 (24.8)	1.60 (0.95–2.67)	0.07
	Self employed	260 (32.0)	159 (61.2)	101 (38.8)	0.83 (0.58–1.17	0.29)
	Student	64 (7.9)	49 (76.6)	15 (23.4)	1.71 (0.91–3.21)	0.09
	Other	108 (13.3)	73 (67.6)	35 (32.4)	1.09 (0.68–1.76)	0.71
**Educational level**	None/primary	213 (26.2)	138 (64.8)	75 (35.2)	ref*	
	Secondary	363 (44.7)	245 (67.5)	118 (32.5)	1.13 (0.79–1.61)	0.51
	Tertiary	236 (29.1)	157 (66.5)	79 (33.5)	1.08 (0.73–1.60)	0.70
**Symptoms (LGTS)**	Discharge curdy/curdled	640 (79.6)	433 (67.7)	207 (32.3)	0.81 (0.57–1.15)	0.24
	Discharge foul-smelling	230 (28.3)	174 (75.7)	56 (24.3)	0.54 (0.39–0.77)	**< 0.001**
	Vulvovaginal itch or pruritus	613 (75.4)	419 (68.4)	194 (31.6)	0.71 (0.51–0.99	**0.04**
	Lower abdominal pain	233 (28.7)	142 (60.9)	91 (39.1)	0.71 (0.52–0.98)	**0.04**
	Vulvovaginal soreness	232 (28.5)	170 (73.3)	62 (26.7)	1.56 (0.46–0.90)	**0.01**
	Dysuria	363 (44.6)	231 (64.6)	132 (36.4)	1.25 (0.94–1.68)	0.13
	Dyspareunia	333 (41.0)	224 (67.3)	109 (32.7)	0.94 (0.70–1.26)	0.67
	Recurrent LGTS^@^	458 (56.3)	276 (60.3)	91 (25.6)	1.91 (1.41–2.59)	**< 0.001**
**Clinical signs**	Abdominal tenderness	29 (3.6)	19 (65.5)	10 (34.5)	1.04 (0.48–2.27)	0.92
	Genital excoriations/ulcers	58 (7.2)	40 (69.0)	18 (31.0)	0.89 (0.50–1.59)	0.70
	Genital erythema/redness	135 (16.7)	92 (68.1)	43 (31.9)	0.92 (0.62–1.37)	0.68
	Genital vesicles	15 (1.9)	12 (80.0)	3 (20.0)	0.49 (0.14–1.76)	0.27
	Genital oedema	38 (4.7)	25 (65.8)	13 (34.2)	1.04 (0.52–2.07)	0.91
	Genital growths/warts	20 (2.5)	15 (75.0)	5 (25.0)	0.66 (0.24–1.83)	0.42
**Contraceptive use in past 12 months**	Contraceptive use∝^1^	528 (64.9)	348 (65.9)	180 (34.1)	0.18 (0.70 − 0.37)	0.67
	Hormonal contraceptive∝^2^	267 (32.8)	177 (66.3)	90 (33.7)	0.99 (0.72–1.35)	0.96
**Antibiotic use in past 4 weeks**		192 (23.6)	129 (67.2)	63 (32.8)	0.96 (0.68–1.35)	0.80
**Antifungal use in past 4 weeks**		83 (10.2)	51 (61.4)	32 (38.6)	1.27 (0.80–2.03)	0.31
**Vaginal practices**^⊗^ **present**		269 (33.1)	190 (70.6)	79 (29.4)	1.33 (0.97–1.83)	0.07
**Parity**	0	203 (25.2)	134 (66.0)	69 (34.0)	ref*	
	1–2	441 (54.3)	290 (65.8)	151 (34.2)	0.59 (0.42–0.83)	**< 0.001**
	3 or more	166 (20.5)	114 (68.7)	52 (31.3)	1.13 (0.73–1.75)	0.59
**Condom use past 3 months**		208 (25.6)	134 (64.4)	74 (35.6)	0.89 (0.64–1.24)	0.48
**Sex partners last 3 months**	0 sex partners	82 (10.1)	50 (61.0)	32 (39.0)	ref*	
	1 sex partner	694 (85.4)	464 (66.9)	230 (33.1)	1.29 (0.81–2.07)	0.29
	2 or more sex partners	37 (4.5)	26 (70.3)	11 (29.7)	1.51 (0.66–3.48)	0.33
**Last sexual contact**	0 to 7 days	356 (46.3)	240 (67.4)	116 (32.6)	ref*	
	8 to 14 days	129 (15.9)	94 (72.9)	35 (27.1)	1.30 (0.83–2.03)	0.25
	More than 14 days	284 (34.9)	182 (64.1)	102 (35.9)	0.86 (0.62–1.20)	0.38

LGTS: Lower genital tract symptoms, NCC: Nairobi City County, CI: confidence interval

^#^The first column in this table uses column percentages to show the distribution of participants over the different variable categories; for the other columns row percentages are used

^¥^Odds for infection

*For variables with multiple categories, the first category was used as reference for the odds ratio

^@^Recurrent LGTS: 3 or more LGTS episodes in preceding 12 months, including the episode during the study visit

∝^1^All contraceptives excluding condoms - hormonal, tubal ligation, intrauterine device, natural/herbal

∝^2^Hormonal contraceptives - oral pills, Norplant, injectables

^⨂^Vaginal practices - douching, use of vaginal inserts

All the participants reported having vaginal discharge. The next most common symptom was vulvovaginal itch (75%), followed by dysuria (45%) and dyspareunia (41%). Experience of three or more LGTS episodes in the preceding 12 months was reported by 56% of the participants. Clinical signs were infrequent, ranging from 2% (genital vesicles) to 17% (vulvovaginal erythema). Recurrence of LGTS and the symptoms of foul-smelling vaginal discharge, vulvovaginal itch, lower abdominal pain, and vulvovaginal soreness were more prevalent in women with a laboratory confirmed infection compared to those without an infection. (Table 1) The distribution of participant characteristics by specific aetiologies are presented in Supplementary Tables S1 – S8.

### Laboratory confirmed aetiologies for LGTS

Of the 813 participants, 540 (66%) had at least one infection. The prevalence of the specific infections was: VVC 40% with 52% of these being RVVC, BV 17%, NG 14%, CT 13%, TV 10%, and MG 6%. Overall, there were 183 (23%) participants with two or more infections, of whom 126 (69%) had dual infections while the rest had multiple (three or more infections), mostly among the STI. (Table 2)

**Table 2:** Laboratory confirmed infections in women presenting with LGTS at outpatient clinics in NCC, Kenya (n = 813)

Infection type	Overall* n (%) 813 (100)	Single infection n (%)	Dual infections n (%)	≥ 3 infections n (%)	Combinations in the dual infections					
					**MG**	**NG**	**CT**	**TV**	**BV**	**VVC**
**Vulvovaginal Candidiasis (VVC)**	325 (40.0)	207 (63.7)	84 (25.8)	34 (10.5)	7	19	17	14	27	207
**Bacterial vaginosis (BV)**	137 (17.1)	57 (41.6)	52 (38.0)	28 (20.4)	4	6	10	5	57	
**Trichomonas vaginalis (TV)**	76 (9.8)	27 (35.5)	27 (35.5)	22 (29.0)	0	7	1	27		
**Chlamydia trachomatis (CT)**	97 (12.5)	21 (21.6)	37 (38.1)	39 (40.2)	2	7	21			
**Neisseria gonorrhoea (NG)**	111 (14.3)	39 (35.1)	39 (35.1)	33 (29.7)	0	39				
**Mycoplasma genitalium (MG)**	43 (5.6)	6 (14.0)	13 (30.2)	24 (55.8)	6					
**Total**	540 (66.4)	357 (66.1)	126 (23.3)	57 (10.6)	13	39	37	27	52	84

*The first column in this table uses column percentages to show the overall distribution of the various infections; for the other columns row percentages are used. Denominator for each infection is less by the respective missed tests. Missing laboratory tests per aetiology were: BV 12, TV 39, NG 39, CT 39, MG 39

LGTS: Lower genital tract symptoms

NCC: Nairobi City County

### Predictors for the aetiologies

The multivariate logistic regression analysis showed that only vulvovaginal itch was associated with the diagnosis VVC (OR 2.20, 95% CI 1.40–3.46). A foul-smelling vaginal discharge was the predictor for BV (OR 3.63, 95% CI 2.17–6.07), while dysuria (OR 0.46, 95% CI 0.27–0.80) and dyspareunia (OR 0.46, 95% CI 0.26–0.82) were negatively predictive of BV. The predictors of any STI were a foul-smelling vaginal discharge (OR 1.64, 95% CI 1.06–2.55), and lower abdominal pain (OR 1.73, 95% CI 1.07–2.79); while recurrent LGTS episodes was negatively predictive of STI (OR 0.45, 95% CI 0.29–0.68). For individual STI, TV was predicted by having a foul-smelling vaginal discharge (OR 2.49, 95% CI 1.29–4.82) and lower abdominal pain (OR 3.02, 95% CI 1.23–7.42); a low level of education was negatively predictive of NG (OR 0.50, 95% CI 0.26–0.95); recurrent LGTS episodes was negatively predictive of CT (OR 0.44, 95% CI 0.25–0.81) and NG (OR 0.46, 95% CI 0.27–0.76); and condom use in the previous 3 months was negatively predictive of MG (OR 0.39, 95% CI 0.17–0.91). Lastly recurrent LGTS episodes was predictive of absence of an infection (OR 2.00, 95% C1.32-3.13); while having foul-smelling vaginal discharge (OR 0.46, 95% CI 0.29–0.74), and lower abdominal pain (OR 0.62, 95% CI 0.39–0.97) were negatively predictive of absence of infection. (Table 3 and Supplementary tables S9 -S16).

**Table 3:** Statistical associations between participant characteristics and aetiologies in multivariate logistic regression (n = 507)

Aetiology	Characteristic	Number of participants n^*^ (%)		OR (95% CI)	p-value
		With the aetiology n (%)	Without the aetiology n (%)		
**VVC**	Total	204 (100.0)	303 (100)		
	Itch or pruritus	172 (84.3)	215 (71.0)	2.20 (1.40–3.46)	< 0.001
**BV**	Total	94 (100%)	406 (100)		
	Foul smell	48 (51.1)	106 (26.1)	3.63 (2.17–6.07)	< 0.001
	Dysuria	28 (29.8)	206 (50.7)	0.46 (0.27–0.80)	0.01
	Dyspareunia	25 (26.6)	185 (45.6)	0.46 (0,26-0.82)	0.01
**Any STI**	Total	136 (100.0)	347 (100.0)		
	Foul smell discharge	45 (33.1)	104 (30.0)	1.64 (1.06–2.55)	0.03
	LAP	106 (77.9)	242 (69.7)	1.73 (1.07–2.79)	0.03
	Recurrent LGTS^@^	59 (43.4)	211 (60.8)	0.45 (0.29–0.68)	< 0.001
TV	Total	53 (100.0)	430 (100.0)		
	Foul smell discharge	27 (50.9)	122 (28.4)	2.49 (1.29–4.82)	0.01
	LAP	44 (83.0)	304 (70.7)	3.02 (1.23–7.42)	0.02
CT	Total	62 (100.0)	421 (100.0)		
	Recurrent LGTS^@^	26 (41.9)	244 (58.0)	0.44 (0.25–0.81)	0.01
NG	Total	79 (100.0)	404 (100.0)		
	≤ Primary education	15 (19.0)	109 (27.0)	0.50 (0.26–0.95)	0.03
	Recurrent LGTS^@^	33 (41.8)	237 (58.7)	0.46 (0.27–0.76)	< 0.001
MG	Total	28 (100.0)	455 (100.0)		
	Condom use^†^	12 (42.9)	122 (26.8)	0.39 (0.17–0.91)	0.03
**None**	Total	165 (100.0)	342 (100.0)		
	Foul smell discharge	34 (20.6)	121 (35.4)	0.46 (0.29–0.74)	< 0.001
	LAP	55 (33.3)	88 (25.7)	0.62 (0.39–0.97)	0.04
	Recurrent LGTS^@^	108 (65.5)	177 (51.8)	2.00 (1.32–3.13)	< 0.001

VVC - Vulvovaginal candidiasis; BV - Bacterial vaginosis; STI – Sexually transmitted infection; TV - Trichomonas vaginalis; NG - Neisseria gonorrhoea; CT - Chlamydia trachomatis; MG - Mycoplasma genitalium

LAP – Lower abdominal pain; LGTS: Lower genital tract symptoms; OR: Odds ratio; CI: Confidence interval

*Denominator for each infection is less by the respective missed tests

^@^Recurrent LGTS: 3 or more LGTS episodes in preceding 12 months, including the episode during the study visit

^†^Condom use: Condom use in preceding 3 months

### Development of an alternative algorithm

**Fig. 2:**
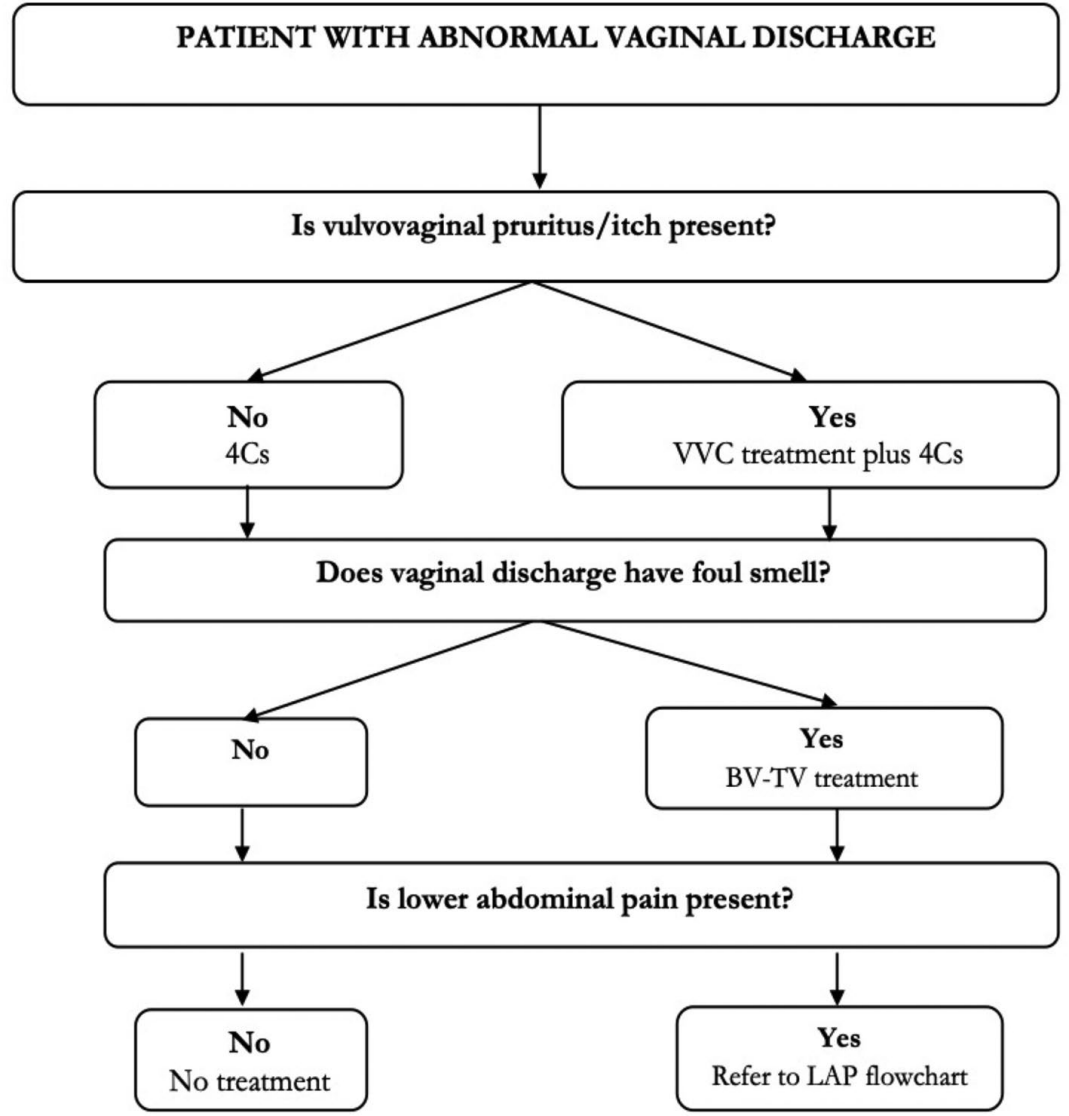
Alternative algorithm for management of vaginal discharge syndrome in Kenya Legend: VVC - Vulvovaginal candidiasis; BV - Bacterial vaginosis; TV - Trichomonas vaginalis VVC treatment = Antifungal (intravaginal or oral) BV-TV treatment = Metronidazole; LAP: Lower abdominal pain 4Cs: Counseling, Compliance, Condom use and Contact tracing

Applying the predictors from the multivariate logistic regression analyses, we developed a flowchart (alternative algorithm) categorizing patients for treatment. Starting with the most prevalent infection, vulvovaginal itch was applied to identify patients for VVC treatment, next foul-smelling vaginal discharge was applied to identify patients for BV-TV treatment, and finally lower abdominal pain was used to identify patients with CT, NG, MG for referral to LAP flowchart. Then patients with no vulvovaginal itch, foul-smelling discharge and lower abdominal pain were categorized for no treatment (Fig. 2).

### Performance of the current and alternative algorithms in the diagnosis and treatment of LGTI

#### Classification of participants by treatment category

From the 306 validation participants the treatment categories by laboratory testing were 121 (40%) VVC, 66 (22%) BV-TV, and 64 (21%) LAP. By the current syndromic algorithm 68% (n = 209) of the 306 validation participants were classified into vaginitis treatment category (VVC plus BV- TV), while the rest (32%, n = 97) were classified into the LAP referral category. The current algorithm’s ability to classify individuals into no treatment was – by definition – nil. The alternative algorithm classified participants into the treatment categories as follows: − 74% (n = 226) VVC, 23% (n = 71) BV-TV, and 28% (n = 86) LAP referral; in addition, 11% were classified into no treatment category. McNemar test showed that the treatment classification accuracy by the current and alternative algorithms for the categories VVC, BV-TV and No treatment differed significantly; p = 0.04, p = 0.02 and p < 0.001 respectively. The classification for LAP treatment did not differ between the two algorithms, p = 0.5. (Table 4 and Supplementary table S17)

**Table 4:** Treatment allocation for LGTI/syndrome, by the current and alternative algorithms (n = 306)

Treatment category (n)	Correct treatment n (% of those with the infection)		Missed treatment n (% of those with the infection)		Inappropriate treatment n (% of those without the infection)		χ², p-value (McNemar)
	Current algorithm	Alternative algorithm	Current algorithm	Alternative algorithm	Current algorithm	Alternative algorithm	
**VVC (121)**	89 (73.6)	102 (84.3)	32 (26.4)	19 (15.7)	120 (64.9)	124 (67.0)	4.11, 0.04
**BV-TV (66)**	45 (68.2)	31 (47.0)	21 (31.8)	35 (53.0)	164 (68.3)	40 (16.7)	5.63, 0.02
**LAP (64)**	22 (34.4)	20 (31.3)	42 (65.6)	44 (68.8)	75 (31.0)	66 (27.3)	0.5, 0.5
**No treatment (108)**	0 (0.0) *	15 (13.9) *	108 (100) #	93 (86.1) #	198 (100) ^¥^	93 (47.0) ^¥^	13.07, p < 0.001

McNemar analysis: Comparisons are per correct treatment and missed treatment; inappropriate treatment was (per definition) not included in the analysis

LGTI: Lower genital tract infections

VVC: Vulvovaginal candidiasis

BV-TV: Bacterial vaginosis-Trichomonas vaginalis

LAP: Lower abdominal pain; includes any of Neisseria gonorrhoea, Chlamydia trachomatis, Mycoplasma genitalium

*Participants without an infection that were correctly classified to receive no treatment

# Participants (with an infection) who needed treatment but were incorrectly classified into No treatment

¥ Participants (with no infection) who didn’t require treatment but received it, of those not requiring treatment

#### Algorithms’ accuracy in overall treatment allocation

**Fig. 3:**
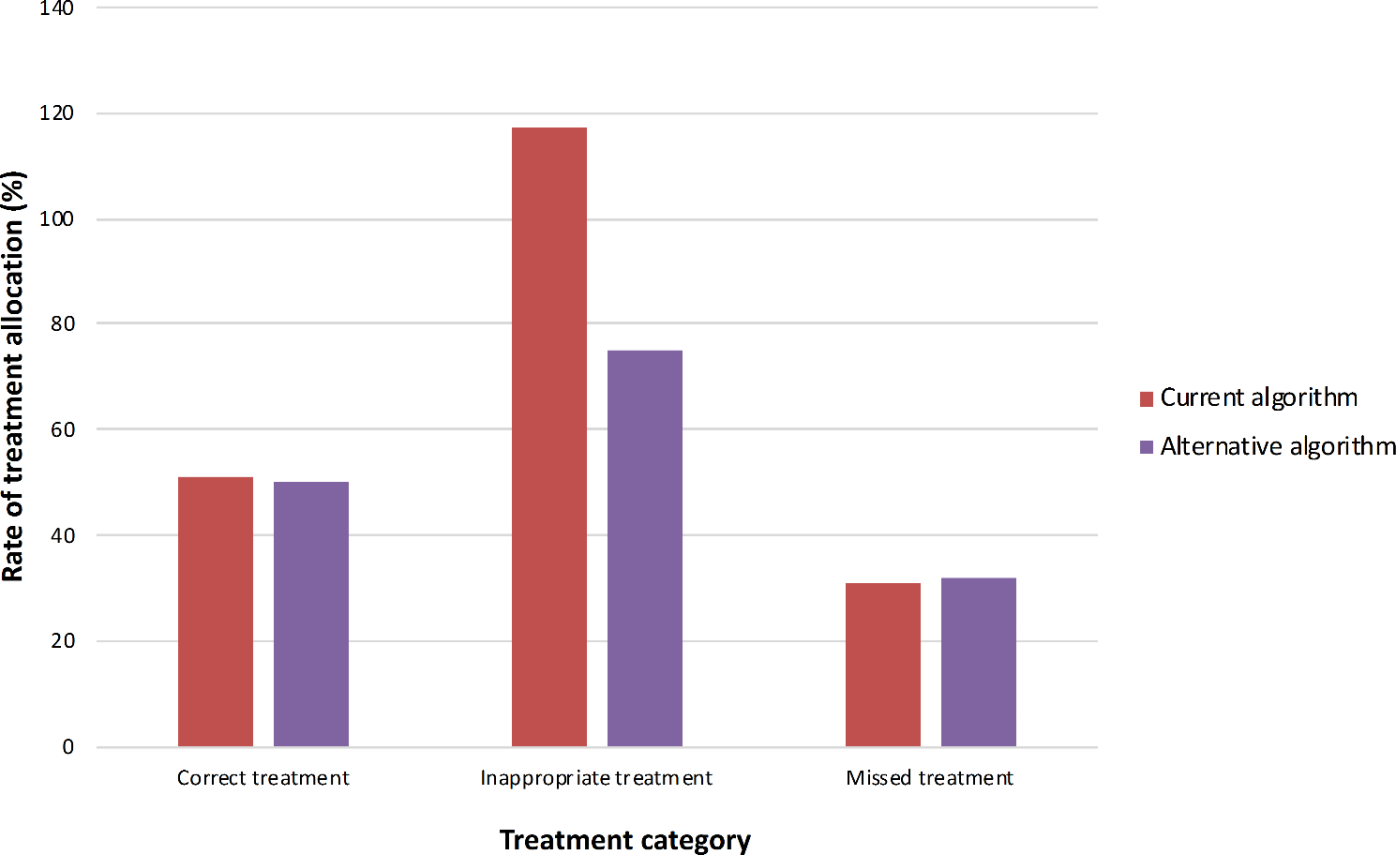
Overall LGTI treatment allocation rates by the current and alternative algorithms (n = 306) Legend: LGTI: Lower genital tract infections Correct treatment: Treatment consistent with the microbiological diagnosis Inappropriate treatment: Irrelevant treatment with reference to the laboratory test results Missed treatment: The necessary treatment not recommended despite laboratory-confirmed infection

By the current algorithm, the overall rate of correct treatment was 51% (n = 156), inappropriate treatment was 117% (n = 359), while missed treatment was 31% (n = 95). The rates by the alternative algorithm were 50% (n = 153) correct treatment, 75% (n = 230) inappropriate treatment, and 32% (n = 98) missed treatment. (Fig. 3)

### Algorithms’ accuracy in specific treatment category allocation

By the current algorithm, 74%, 68%, 34% and 0% of participants with VVC, BV-TV, LAP, and No infection respectively, were correctly treated, while by the alternative algorithm 84%, 47%, 31%, and 14% of participants with VVC, BV-TV, LAP and no infection respectively would get correct treatment. Inappropriate treatment rates by the current algorithm were 65%, 68% and 31% for VVC, BV-TV and LAP respectively, and 100% for the ‘no infection’ group; while by the alternative algorithm inappropriate treatment was 67%, 17%, and 27% for VVC, BV-TV and LAP respectively, and 47% for the no infection group. Failure to give the required treatment was lowest for VVC and highest for LAP group, by both algorithms. (Table 4)

### Performance scores in treatment allocation

The performance scores in treatment allocation for VVC treatment by both algorithms were similar i.e. high sensitivity (> 73%), moderate NPV (> 66%), and low specificity and PPV (33-45%). For BV-TV treatment, the specificity by the current algorithm was curiously low (32%), but notably high by the alternative algorithm (82%); the NPV for BV-TV treatment was 78% and 85% by the current and alternative algorithms respectively. For LAP referral the performance of both algorithms was similar: - low sensitivity (< 35%), low PPV (< 24%), moderate specificity (about 70%), and a fairly high NPV (80%). With regard to no treatment, the alternative algorithm had a specificity of 91%, a moderate NPV of 66%, but a poor sensitivity of 14%.

Overall, the alternative algorithm performed better than the current algorithm. This was especially notable in BV-TV treatment - accuracy scores of 74% compared to 40%; and in allocation into No treatment, accuracy of 64% versus 0%. (Table 5)

**Table 5:** Performance scores in LGTI treatment allocation by the current and alternative algorithms

	VVC treatment		BV-TV treatment		LAP treatment*		No Treatment	
	Current algorithm	Alternative algorithm	Current algorithm	Alternative algorithm	Current algorithm	Alternative algorithm	Current algorithm	Alternative algorithm
Statistic	Value (95% CI)	Value (95% CI)	Value (95% CI)	Value (95% CI)	Value (95% CI)	Value (95% CI)	Value (95% CI)	Value (95% CI)
Sensitivity (%)	73.6 (64.8–81.2)	84.3 (76.6–90.3)	68.2 (55.6–79.1)	47.0 (34.6–59.7)	34.4 (23.0-47.3)	31.3 (20.2–44.1)	-	13.9 (8.0-21.9)
Specificity (%)	35.1 (28.3–42.5)	33.0 (26.3–40.3)	31.7 (25.8–38.0)	81.7 (76.2–86.4)	69.0 (62.8–74.8)	71.1 (64.9–76.7)	-	90.9 (86.0-94.5)
PPV (%)	42.6 (35.8–49.6)	45.1 (38.5–51.9)	21.5 (16.2–27.7)	41.3 (30.1–53.3)	22.7 (14.8–32.3)	22.2 (14.1–32.2)	-	45.5 (28.1–63.7)
NPV (%)	67.0 (56.7–76.2)	76.3 (65.4–85.1)	78.4 (68.8–86.1)	84.9 (79.6–89.2)	79.9 (73.8–85.1)	79.6 (73.6–84.8)	-	65.9 (60.0-71.5)
Accuracy (%)	50.3 (44.6–56.1)	53.3 (47.5–59.0)	39.5 (34.0-45.3)	74.2 (68.9–79.0)	61.8 (56.1–67.2)	62.8 (57.1–68.2)	-	63.7 (58.1–69.1)

LGTI: Lower genital tract infections, VVC: Vulvovaginal candidiasis, BV-TV: Bacterial vaginosis-Trichomonas vaginalis, LAP: Lower abdominal pain; includes any of Neisseria gonorrhoea, Chlamydia trachomatis, and Mycoplasma genitalium

VVC treatment = Antifungal (intravaginal or oral)

BV-TV treatment = Metronidazole

*LAP treatment = Referral to LAP syndromic treatment algorithm

PPV - Positive Predictive Value

NPV - Negative Predictive Value

## Discussion

Two-thirds of the women presenting with LGTS at outpatient clinics in Nairobi had at least one confirmed LGTI, more than one third of the infected had multiple infections, and a majority reported at least 3 episodes in a year. VVC was the most frequent infection (2 of 5 women) while BV prevalence was remarkably low. Symptoms of vaginitis were predominant in these mostly young women, but clinical signs were scanty. Contraceptive use was high; condom use was low, in line with a predominance of reported monogamous relationships. The vaginal discharge syndrome algorithm used in Kenya proved to be insufficient for the management of genital infections, but our proposed alternative achieved only modest improvement.

The frequencies of specific genital infections in our study vary from rates detected previously at similar clinics in Nairobi in which VVC was 6% higher and TV was double, but NG and CT rates were lower [22]. With vaginal discharge and itch being the commonest clinical presentations and coupled with recurrent symptoms, we speculate that a high usage of vaginitis (VVC and BV-TV) treatment with delayed opportunity for STI treatment over the years may explain these variations. Our detection of an infection in two-thirds of patients is similar to proportions in studies from elsewhere in Africa [8, 9], but somewhat lower than studies from India (80%) [7]. For the specific female genital infections, our findings do not concur with other studies from Africa where BV was predominant [2, 8, 9, 17]. These variations are likely due to study population differences. Indeed, we noted associations between patient characteristics and the infections. Influence by study population characteristics such as sexual risk behaviour, level of education, age, condom use, prior use of antimicrobials, and perhaps genetics have been cited as determinants of aetiology in other studies [3, 8, 9, 17, 23].

About one-third of the patients in our study tested negative to the six common LGTI despite being symptomatic. We think that the probability of false negative test results due to prior antimicrobial use is small because we used very sensitive testing methods. We employed PCR for detection of the four STIs; this technique would identify even antimicrobial-suppressed bacteria and TV. For Candida infection we employed 3 techniques i.e., KOH, gram stain and culture, hence the possibility of false negative cases was low. For bacterial vaginosis we used the Nugent score, which is the gold standard.

Although the vaginal discharge syndrome tool has the advantage of providing treatment to patients at an opportune time and without the laboratory-testing-associated delays and costs, we identified concerning discrepancies between the syndromic predictions and actual infections. Hence, we sought to improve the algorithm’s accuracy by determining patient characteristics more predictive of the infections. The symptoms of vulvovaginal itch for VVC and repulsive vaginal discharge for BV-TV were crucial in delineating the vaginitis syndrome to guide specific treatment for VVC and BV-TV, so as to reduce the over-use of metronidazole given the disparate burden of VVC and BV-TV. It is however worth noting that although important for detection of STI, LAP was an infrequent symptom hence contributing to the low sensitivity and PPV by both algorithms.

Our study showed no association between contraceptive use (including hormonal) and STI in general or with specific STI. Although controversial, studies in the past have pointed toward higher likelihood of some STI in clients using hormonal contraceptives. Indeed, a recent systematic review and meta-analysis on studies investigating the influence of hormonal contraceptives on STI reports mixed findings that included no effect, a protective effect, and increased risk [24].

The significant association between symptom recurrence and absence of infection was unexpected, especially because we employed DNA detection for most microbes. We speculate that, in addition to the effects of prior antimicrobial use in almost one quarter our participants, vaginal pathobionts (not tested in our study) could partly explain this; additionally women may not be able to distinguish between physiologic and abnormal leucorrhoea, hence overreport vaginal discharge as has been shown elsewhere [25]. Future studies are necessary, to elucidate this.

A syndromic-only approach can be misleading as a diagnosis and treatment tool. Indeed, we demonstrate here that the vaginal discharge syndrome algorithm used in Kenya is poor at detecting or excluding infections. With the algorithm’s low specificity and PPV, about two-thirds of patients in our study received unnecessary metronidazole and antifungal treatment, while a similar proportion of patients requiring treatment for bacterial STI did not receive it. Both algorithms had low sensitivity and poor PPV scores for STI. Our findings are in line with other studies which have revealed the inadequacies of the syndromic flowcharts in diagnosis and treatment of female genital infections [18, 19, 26]. Our substitute algorithm had advantages over the current algorithm. By avoiding blanket treatment of VVC, BV and TV, our algorithm performed better for BV-TV treatment by lowering the unnecessary use of metronidazole, and somewhat for VVC treatment too. Our substitute algorithm also recognized women without infection leading to less overtreatment.

Vaginal discharge-based syndromic approaches have been shown in the past, and confirmed in this study, to miss common bacterial STI. This however should not motivate for inclusion of bacterial STI treatment to these algorithms. Such a move would result in unnecessary use of antibiotics in three-quarters of STI-free patients, posing the risk of development of antimicrobial resistance. We rather advocate that the savings from such unnecessary antibiotic prescriptions instead be channelled to point-of-care (POC) testing costs for patients triaged to have STI by the syndromic algorithm. A broad interrogation of the syndromic approach, beyond accuracy in treatment allocation, is needed to determine the full value of our proposal.

Being symptom-dependent, the syndromic flowcharts are poor at detecting mixed infections, yet we found this to be common, particularly with STI whose mode of transmission and clinical presentation are shared. Several studies have shown coupling of some infections, especially TV with BV and with bacterial STI [27–29]; improved/future algorithms should thus take this into consideration. Moreover, the symptom-dependent approaches do not recognize the existence of asymptomatic infections, yet studies show that up to 80% of patients with TV or BV are asymptomatic [2, 30]; these patients remain unrecognized and not treated in the symptom-dependent algorithms.

Efforts by others elsewhere to improve the syndromic algorithm’s performance have yielded limited improvement. Such attempts included addition of sexual partner risk behavior information, and bedside tests e.g. vaginal swab pH and whiff test [17, 31–33]. The problems are that different etiologies share similar clinical characteristics and certain patients lack certain symptoms and signs despite having the disease; additionally, symptoms are largely subjective. For example, vulvovaginal itch is more common in women with VVC, but a large proportion of women with other infections also have it; and foul-smelling vaginal discharge is associated with BV, TV and STI [17, 26, 34, 35]. Hence the algorithm’s performance is limited by indistinguishable behavioral factors, and symptoms and signs. The result of this is a suboptimal sensitivity and specificity. Therefore, only with integration of POC into the algorithms is good discriminative power achievable.

POC testing is feasible and accepted by women [36]. Such tests would be crucial in delineating mixed infections, asymptomatic infections or deciphering infections with shared symptom(s). For example, inclusion of POC pH and biochemical testing, for BV and TV respectively, yields notable improvement in diagnostic accuracy [17]. Additionally, several studies show that real-time PCR testing for STI is very promising with high sensitivity and specificity. These rapid and accurate tests are relatively affordable, making it possible to implement them in resource-limited settings. For such settings, a combination of syndromic triage plus POC testing would be best suited [37–39]. However, given the long-standing funding gaps in the public sector in these settings, widespread use of POC is unlikely to be realized in the immediate future. Therefore, while use of the syndromic approach remains the most feasible option, regular review and revision of the algorithms’ performance in line with emerging evidence is vital. Relatedly, it is necessary to rethink the present approaches to algorithm evaluation; they are limited to diagnostic and treatment accuracy. We propose that future evaluations of algorithms be comprehensive and include short-term and long-term opportunity costs determinations, and cost-benefit analyses.

A limitation of our study is that a significant number of patients had multiple infections, and our analyses did not look at the influence of multiple infections on the predictors. However, we were able to interrogate the performance of the conventional vaginal discharge syndrome treatment flowchart using a large dataset. Our sizeable dataset additionally allowed us to subject our alternative algorithm to internal validation. Secondly, our study did not access patients who seek care at private healthcare facilities. We however believe that our study population is representative of women in Nairobi. While it is expected that patients of lower socioeconomic status would seek health care mainly from public health facilities, it has been shown that only one-third of patients from a slum in Nairobi seek care at public health services with a majority utilizing private facilities [40]. Notably, 43% of people in informal settlements in Nairobi have health insurance cover compared to a national proportion of 20% [41, 42]. Moreover, key bio-behavioral characteristics of the participants such as age, marital status etc., do not vary between those who use public and private facilities and therefore the prevalence and type of LGTS is not expected to vary.

## Conclusion

Most symptomatic women had a genital infection including multiple infections, yet the algorithm in use was largely inadequate in offering the required treatment. A significant proportion of patients therefore were not given the correct treatment, but many also received unnecessary antimicrobials. This is the first time in Kenya that the performance of the syndromic algorithm presently used in the management of vaginitis has been interrogated, and an improved flowchart proposed and validated. Our alternative algorithm provides only modest improvement, especially in reducing the inappropriate use of metronidazole. For timely and optimum management of genital tract infections in women, we recommend a combination of syndromic triage and POC testing.

### Electronic supplementary material

Below is the link to the electronic supplementary material.

**Supplementary tables and Figures: Table S1**: Socio-demographic and clinical characteristics in vulvovaginal candidiasis (VVC) positive and negative cases. **Table S2**: Socio-demographic and clinical characteristics in bacterial vaginosis (BV) positive and negative cases. **Table S3**: Socio-demographic and clinical characteristics in any STI (TV, MG, CT, NG) positive and negative cases. **Table S4**: Socio-demographic and clinical characteristics in Trichomonas vaginalis (TV) positive and negative cases. **Table S5**: Socio-demographic and clinical characteristics in Neisseria Gonorrhoea (NG) positive and negative cases. **Table S6**: Socio-demographic and clinical characteristics in Chlamydia trachomatis (CT) positive and negative cases. **Table S7**: Socio-demographic and clinical characteristics in Mycoplasma genitalium (MG) positive and negative cases. **Table S8**: Prevalence of symptoms for no infection and any infection cases. **Table S9**: Multivariate binary logistic regression (last step) vulvovaginal candidiasis. **Table S10:** Multivariate binary logistic regression (last step) Bacterial vaginosis. **Table S11**: Multivariate binary logistic regression (last step) Any STI. **Table S12**: Multivariate binary logistic regression (last step) Trichomonas vaginalis. **Table S13**: Multivariate binary logistic regression (last step) Chlamydia trachomatis. **Table S14**: Multivariate binary logistic regression (last step) Neisseria Gonorrhoea. Table S15: Multivariate logistic regression (last step) Mycoplasma genitalium. **Table S16**: Multivariate logistic regression (last step) no infection. **Table S17**: McNemar test - computation comparing treatment allocation for LGTI/syndrome, by the current and alternative algorithms (n=306)

## Data Availability

The datasets used and/or analyzed during the current study are available from the corresponding author on reasonable request.

## List of abbreviations

BV: Bacterial vaginosis
CI: Confidence interval
CT: Chlamydia trachomatis
LAP: Lower abdominal pain
LGTI: Lower genital tract infections
LGTS: Lower genital tract symptoms
MG: Mycoplasma genitalium
NCC: Nairobi City County
NG: Neisseria gonorrhoea
NPV: Negative predictive value
OR: Odds ratio
PCR: Polymerase chain reaction
POC: Point-of-care
PPV: Positive predictive value
RVVC: Recurrent vulvovaginal candidiasis
STI: Sexually transmitted infections
TV: Trichomonas vaginalis
VVC: Vulvovaginal candidiasis
WHO: World Health Organization
