# Supplementary material for: Evaluation and optimization of the syndromic management of female genital tract infections in Nairobi, Kenya
Source: BMC Infect Dis. 2023 Aug 22;23:547. doi: 10.1186/s12879-023-08442-2 (PMC10463802; doi:10.1186/s12879-023-08442-2)
Supplement: Supplementary file 1 — Supplementary tables and Figures: Table S1: Socio-demographic and clinical characteristics in vulvovaginal candidiasis (VVC) positive and negative cases. Table S2: Socio-demographic and clinical characteristics in bacterial vaginosis (BV) positive and negative cases. Table S3: Socio-demographic and clinical characteristics in any STI (TV, MG, CT, NG) positive and negative cases. Table S4: Socio-demographic and clinical characteristics in Trichomonas vaginalis (TV) positive and negative cases. Table S5: Socio-demographic and clinical characteristics in Neisseria Gonorrhoea (NG) positive and negative cases. Table S6: Socio-demographic and clinical characteristics in Chlamydia trachomatis (CT) positive and negative cases. Table S7: Socio-demographic and clinical characteristics in Mycoplasma genitalium (MG) positive and negative cases. Table S8: Prevalence of symptoms for no infection and any infection cases. Table S9: Multivariate binary logistic regression (last step) vulvovaginal candidiasis. Table S10: Multivariate binary logistic regression (last step) Bacterial vaginosis. Table S11: Multivariate binary logistic regression (last step) Any STI. Table S12: Multivariate binary logistic regression (last step) Trichomonas vaginalis. Table S13: Multivariate binary logistic regression (last step) Chlamydia trachomatis. Table S14: Multivariate binary logistic regression (last step) Neisseria Gonorrhoea. Table S15: Multivariate logistic regression (last step) Mycoplasma genitalium. Table S16: Multivariate logistic regression (last step) no infection. Table S17: McNemar test - computation comparing treatment allocation for LGTI/syndrome, by the current and alternative algorithms (n=306) [file 12879_2023_8442_MOESM1_ESM.docx]

**Supplementary tables and Figures**

**Table S1**: Socio-demographic and clinical characteristics in vulvovaginal candidiasis (VVC) positive and negative cases

|  | Candida positive n (%)  n = 325 | No candida n (%)  n = 488 | OR (95% CI, p-value) |
| --- | --- | --- | --- |
| Demographics | | | |
| Age  18 to 25 years  26 to 35 years  36 and above | 119 (37.4)  141 (44.3)  58 (18.2) | 187 (39.0)  184 (38.4)  108 (22.5) | ref*  0.83 (0.60-1.14, 0.25)  1.19 (0.80-1.76, 0.40) |
| Educational level  None/primary  Secondary  Tertiary | 83 (25.5)  143 (44.0)  99 (30.5) | 130 (26.7)  220 (45.2)  137 (28.1) | ref*  0.98 (0.70-1.39, 0.92)  0.88 (0.61-1.29, 0.52) |
| Marital status  Single  Married  Separated, divorced or widowed | 109 (33.5)  186 (57.2)  30 (9.2) | 162 (33.3)  259 (53.2)  66 (13.6) | ref*  0.94 (0.69-1.27, 0.68)  1.48 (0.90-2.43, 0.12) |
| Occupation  Unemployed, housewife  Professional worker  Self employed  Student  Other | 110 (33.8)  45 (13.8)  103 (31.7)  28 (8.6)  39 (12.0) | 169 (34.7)  56 (11.5)  157 (32.2)  36 (7.4)  69 (14.2) | ref* |
| Ethnicity  Other  Luhya  Kikuyu  Kamba | 72 (22.4)  69 (21.4)  129 (40.1)  52 (16.1) | 126 (26.0)  98 (20.2)  192 (39.6)  69 (14.2) | ref*  0.82 (0.53-1.24, 0.33)  0.85 (0.59-1.23, 0.39)  0.76 (0.48-1.20, 0.24) |
| Symptoms |  |  |  |
| Discharge curdy/curdled | 269 (83.0) | 371 (77.3) | 1.44 (1.00-2.06, **0.05**) |
| Discharge foul smell | 91 (28.0) | 139 (28.5) | 0.98 (0.72-1.33, 0.88) |
| Vulvar itch or pruritus | 274 (84.3) | 339 (69.5) | 2.36 (1.66-3.37, **0.00**) |
| Lower abdominal pain | 83 (25.5) | 150 (30.7) | 0.77 (0.56-1.06, 0.11) |
| Soreness | 110 (33.8) | 122 (25.0) | 1.54 (1.13-2.09, **0.01**) |
| Erythema redness | 58 (17.9) | 77 (16.0) | 1.15 (0.79-1.67, 0.47) |
| Dysuria | 133 (40.9) | 230 (47.1) | 0.78 (0.59-1.03, 0.08) |
| Dyspareunia | 147 (45.2) | 186 (38.1) | 1.34 (1.01-1.78, **0.04**) |
| Recurrent LGTS previous 12 months | 168 (51.7) | 290 (59.4) | 0.73 (0.55-0.97, **0.03)** |
| Signs |  |  |  |
|  |  |  |  |
| Abdominal tenderness | 10 (3.1) | 19 (3.9) | 0.78 (0.36-1.71, 0.54) |
| Excoriations, ulcers or skin lesions | 23 (7.1) | 35 (7.3) | 0.98 (0.57-1.69, 0.93) |
| Vesicles | 8 (2.5) | 7 (1.5) | 1.72 (0.62-4.79, 0.30) |
| Oedema | 18 (5.6) | 20 (4.1) | 1.36 (0.71-2.61, 0.36) |
| Growth warts | 12 (3.7) | 8 (1.7) | 2.28 (0.92-5.64, 0.07) |
| Use of contraceptives | 214 (65.8) | 314 (64.3) | 1.07 (0.796-1.435, 0.66) |
| Contraceptives natural/herbal | 21 (6.5) | 29 (5.9) | 1.09 (0.61-1.95, 0.76) |
| Contraceptives hormonal | 100 (30.8) | 167 (34.2) | 0.85 (0.63-1.15, 0.31) |
| Contraceptives IUCD | 47 (14.5) | 49 (10.0) | 1.52 (0.99-2.32, 0.06) |
| Contraceptives tubal ligation | 3 (0.9) | 3 (0.6) | 1.51 (0.30-7.51, 0.62) |
| Vaginal practices | 109 (33.5) | 160 (32.8) | 1.03 (0.77-1.39, 0.83) |
| Parity  0  1-2  3 or more | 91 (28.1)  158 (48.8)  75 (23.1) | 112 (23.0)  283 (58.2)  91 (18.7) | ref*  1.46 (1.04-2.04, **0.03**)  0.99 (0.65-1.49, 0.95) |
| Sexual behaviour |  |  |  |
| Condom use last 3 months | 78 (24.0) | 130 (26.6) | 1.15 (0.83-1.59, 0.40) |
| Number of sexual partners previous 3 months  0 partners  1 partner  2 or more partners | 29 (8.9)  283 (87.1)  13 (4.0) | 53 (10.9)  411 (84.2)  24 (4.9) | ref*  0.80 (0.49-1.28, 0.35)  1.01 (0.45-2.28, 0.98) |
| Number of sexual partners previous 12 months  0 partners  1 partner  2 or more partners | 10 (3.1)  262 (80.6)  53 (16.3) | 26 (5.3)  378 (77.5)  84 (17.2) | ref*  0.56 (0.26-1.17, 0.12)  0.61 (0.27-1.37, 0.23) |
| Days since last sexual contact  0 to 7 days  8 to 14 days  More than 14 days | 139 (45.0)  59 (19.1)  111 (35.9) | 217 (47.2)  70 (15.2)  173 (37.6) | ref*  0.76 (0.51-1.14, 0.19)  1.00 (0.73-1.37, 0.99) |
| Allergies | 22 (6.8) | 32 (6.6) | 1.04 (0.59-1.82, 0.91) |
| BMI  Under/normal weight  Overweight  Obesity | 155 (52.4)  100 (33.8)  41 (13.9) | 232 (50.1)  156 (33.7)  75 (16.2) | ref*  1.04 (0.75-1.44, 0.80)  1.22 (0.79-1.88, 0.36) |
| Medication use previous 4 weeks |  |  |  |
| Antibiotics | 89 (27.4) | 103 (21.1) | 1.41 (1.02-1.95, **0.04**) |
| Antifungals | 30 (9.2) | 53 (10.9) | 0.84 (0.52-1.34, 0.45) |
| Steroids | 11 (3.4) | 16 (3.3) | 1.03 (0.47-2.26, 0.93) |
| None | 184 (55.6) | 290 (59.4) | 0.89 (0.67-1.18, 0.43) |

**Legend:** *The OR is calculated for the symptom category ‘yes’ compared to ‘no’, unless stated otherwise. e.g. in the case of a symptom with more than two categories, then the first category was used as a reference.

**Table S2:** Socio-demographic and clinical characteristics in bacterial vaginosis (BV) positive and negative cases

|  | BV positive n (%)  n = 137 | No BV n (%)  n = 664 | OR (95% CI, p-value) |
| --- | --- | --- | --- |
| Demographics | | | |
| Age  18 to 25 years  26 to 35 years  36 and above | 61 (46.2)  47 (35.6)  24 (18.2) | 239 (36.6)  275 (42.1)  139 (21.3) | ref*  1.49 (0.98-2.27, 0.06)  1.48 (0.88-2.48. 0.14) |
| Educational level  None/primary  Secondary  Tertiary | 35 (25.5)  62 (45.3)  40 (29.2) | 174 (26.2)  297 (44.8)  192 (29.0) | ref*  0.96 (0.61-1.52, 0.87)  0.97 (0.59-1.59, 0.89) |
| Marital status  Single  Married  Separated, divorced or widowed | 59 (43.1)  56 (40.9)  22 (16.1) | 210 (31.7)  379 (57.2)  74 (11.2) | ref*  1.90 (1.27-2.84, **0.00**)  0.95 (0.54-1.65, 0.84) |
| Occupation  Unemployed, housewife  Professional worker  Self employed  Student  Other | 42 (30.7)  20 (14.6)  37 (27.0)  15 (10.9)  23 (16.8) | 233 (35.1)  77 (11.6)  221 (33.3)  48 (7.2)  84 (12.7) | ref* |
| Ethnicity  Other  Luhya  Kikuyu  Kamba | 28 (20.6)  39 (28.7)  60 (44.1)  9 (6.6) | 169 (25.6)  126 (19.1)  255 (38.6)  110 (16.7) | ref*  0.54 (0.31-0.92, **0.02**)  0.70 (0.43-1.15, 0.16)  2.03 (0.92-4.46, 0.08) |
| Symptoms |  |  |  |
| Discharge curdy/curdled | 108 (79.4) | 523 (79.6) | 0.99 (0.63-1.56, 0.90) |
| Discharge foul smell | 67 (48.9) | 162 (24.4) | 2.97 (2.03-4.33, **0.00**) |
| Vulvar itch or pruritus | 92 (67.2) | 512 (77.1) | 0.61 (0.41-0.91, **0.01**) |
| Lower abdominal pain | 36 (26.3) | 193 (29.1) | 0.87 (0.57-1.32, 0.51) |
| Soreness | 37 (27.0) | 191 (28.8) | 0.92 (0.61-1.39, 0.68) |
| Erythema redness | 18 (13.1) | 114 (17.4) | 0.72 (0.42-1.23, 0.23) |
| Dysuria | 46 (33.6) | 311 (46.8) | 0.57 (0.39-0.84, **0.00**) |
| Dyspareunia | 43 (31.4) | 282 (42.5) | 0.62 (0.42-0.92, **0.02**) |
| Recurrent LGTS previous 12 months | 65 (47.4) | 384 (57.8) | 0.66 (0.45-0.95, **0.03)** |
| Signs |  |  |  |
|  |  |  |  |
| Abdominal tenderness | 6 (4.4) | 23 (3.5) | 1.28 (0.51-3.20, 0.60) |
| Excoriations, ulcers or skin lesions | 11 (8.0) | 43 (6.5) | 1.25 (0.63-2.48, 0.53) |
| Vesicles | 3 (2.2) | 12 (1.8) | 1.20 (0.34-4.32, 0.78) |
| Oedema | 2 (1.5) | 35 (5.3) | 0.26 (0.06-1.11, 0.05) |
| Growth warts | 5 (3.6) | 15 (2.3) | 1.62 (0.58-4.54, 0.35) |
| Use of contraceptives | 89 (65.0) | 430 (64.8) | 1.01 (0.69-1.48, 0.96) |
| Contraceptives natural/herbal | 8 (5.8) | 42 (6.3) | 0.92 (0.42-2.00, 0.83) |
| Contraceptives hormonal | 48 (35.0) | 215 (32.4) | 1.13 (0.77-1.66, 0.55) |
| Contraceptives IUCD | 18 (13.1) | 75 (11.3) | 1.19 (0.69-2.06, 0.54) |
| Contraceptives tubal ligation | 0 (0.0) | 6 (0.9) | 1.21 (1.17-1.25, 0.26) |
| Vaginal practices | 49 (35.8) | 216 (32.5) | 1.16 (0.79-1.70, 0.46) |
| Parity  0  1-2  3 or more | 36 (26.5)  76 (55.9)  24 (17.6) | 163 (24.6)  360 (54.4)  139 (21.0) | ref*  1.05 (0.68-1.62, 0.84)  1.28 (0.73-2.25, 0.39) |
| Sexual behaviour |  |  |  |
| Condom use last 3 months | 32 (23.4) | 174 (26.2) | 0.86 (0.56-1.32, 0.49) |
| Condom use during last sexual contact | 26 (19.0) | 130 (19.6) | 0.96 (0.60-1.54, 0.87) |
| Number of sexual partners previous 3 months  0 partners  1 partner  2 or more partners | 13 (9.5)  112 (81.8)  12 (8.8) | 68 (10.2)  573 (86.3)  23 (3.5) | ref*  0.98 (0.52-1.83, 0.95)  0.37 (0.15-0.92, **0.03**) |
| Number of sexual partners previous 12 months  0 partners  1 partner  2 or more partners | 1 (0.7)  93 (67.9)  43 (31.4) | 34 (5.1)  537 (80.9)  93 (14.0) | ref*  0.17 (0.02-1.26, **0.05**)  0.06 (0.01-0.48. **0.00**) |
| Days since last sexual contact  0 to 7 days  8 to 14 days  More than 14 days | 64 (47.4)  23 (17.0)  48 (35.6) | 285 (45.8)  103 (16.6)  234 (37.6) | ref*  1.01 (0.59-1.70, 0.98)  1.10 (0.73-1.65, 0.67) |
| Allergies | 10 (7.3) | 42 (6.3) | 1.17 (0.57-2.39, 0.67) |
| BMI  Under/normal weight  Overweight  Obesity | 74 (56.5)  39 (29.8)  18 (13.7) | 304 (49.4)  214 (34.7)  98 (15.9) | ref*  1.34 (0.87-2.04, 0.18)  1.33 (0.76-2.33, 0.33) |
| Medication use previous 4 weeks |  |  |  |
| Antibiotics | 17 (12.4) | 169 (25.5) | 0.42 (0.24-0.71, **0.00**) |
| Antifungals | 12 (8.8) | 68 (10.2) | 0.84 (0.44-1.60, 0.60) |
| Steroids | 7 (5.1) | 20 (3.0) | 1.73 (0.72-4.19, 0.22) |

**Legend:** *The OR is calculated for the symptom category ‘yes’ compared to ‘no’, unless stated otherwise. e.g. in the case of a symptom with more than two categories, then the first category was used as a reference.

**Table S3:** Socio-demographic and clinical characteristics in any STI (TV, MG, CT, NG) positive and negative cases

|  | Any STI positive n (%)  n = 249 | No STI n (%)  n = 525 | OR (95% CI, p-value) |
| --- | --- | --- | --- |
| Demographics | | | |
| Age  18 to 25 years  26 to 35 years  36 and above | 112 (45.7)  85 (34.7)  48 (19.6) | 178 (34.7)  223 (43.5)  112 (21.8) | ref*  1.65 (1.17-2.33, **0.00**)  1.47 (0.97-2.22, 0.07) |
| Educational level  None/primary  Secondary  Tertiary | 66 (26.5)  121 (48.6)  62 (24.9) | 138 (26.3)  228 (43.5)  158 (30.2) | ref*  0.90 (0.62-1.30, 0.58)  1.22 (0.81-1.85, 0.35) |
| Marital status  Single  Married  Separated, divorced or widowed | 83 (33.3)  133 (53.4)  33 (13.3) | 171 (32.6)  291 (55.5)  62 (11.8) | ref*  1.06 (0.76-1.48, 0.72)  0.91 (0.56-1.50, 0.72) |
| Occupation  Unemployed, housewife  Professional worker  Self employed  Student  Other | 89 (35.7)  31 (12.4)  62 (24.9)  26 (10.4)  41 (16.5) | 180 (34.4)  62 (11.8)  185 (35.3)  34 (6.5)  63 (12.0) | ref* |
| Ethnicity  Other  Luhya  Kikuyu  Kamba | 59 (24.0)  76 (30.9)  86 (35.0)  25 (10.2) | 130 (24.9)  87 (16.7)  217 (41.6)  88 (16.9) | ref*  0.52 (0.34-0.80, **0.00**)  1.15 (0.77-1.70, 0.50)  1.60 (0.93-2.74, 0.09) |
| Symptoms |  |  |  |
| Discharge curdy/curdled | 189 (76.8) | 421 (81.0) | 0.78 (0.54-1.13, 0.19) |
| Discharge foul smell | 87 (34.9) | 133 (25.3) | 1.58 (1.14-2.19, **0.01**) |
| Vulvar itch or pruritus | 187 (75.1) | 401 (76.4) | 0.93 (0.66-1.33, 0.70) |
| Lower abdominal pain | 61 (24.5) | 160 (30.5) | 0.74 (0.53-1.04, 0.09) |
| Soreness | 69 (27.7) | 150 (28.6) | 0.96 (0.69-1.34, 0.80) |
| Erythema redness | 41 (16.7) | 88 (16.9) | 0.99 (0.66-1.48, 0.95) |
| Dysuria | 120 (48.2) | 227 (43.2) | 1.22 (0.90-1.65, 0.20) |
| Dyspareunia | 96 (38.6) | 221 (42.1) | 0.86 (0.63-1.18, 0.35) |
| Recurrent LGTS previous 12 months | 113 (45.4) | 318 (60.6) | 0.54 (0.39-0.74, **0.00)** |
| Signs |  |  |  |
|  |  |  |  |
| Abdominal tenderness | 10 (4.0) | 17 (3.2) | 1.25 (0.56-2.78, 0.58) |
| Excoriations, ulcers or skin lesions | 12 (4.9) | 44 (8.4) | 0.56 (0.29-1.08, 0.08) |
| Vesicles | 7 (2.8) | 7 (1.3) | 2.16 (0.75-6.21, 0.15) |
| Oedema | 11 (4.5) | 27 (5.2) | 0.86 (0.42-1.76, 0.68) |
| Growth warts | 4 (1.6) | 15 (2.9) | 0.56 (0.18-1.70, 0.30) |
| Use of contraceptives | 162 (65.1) | 341 (65.0) | 1.01 (0.73-1.38, 0.98 |
| Contraceptives natural/herbal | 17 (6.8) | 31 (5.9) | 1.17 (0.63-2.15, 0.62) |
| Contraceptives hormonal | 85 (34.1) | 169 (32.2) | 1.09 (0.79-1.50, 0.59) |
| Contraceptives IUCD | 25 (10.0) | 68 (13.0) | 0.75 (0.46-1.22, 0.24) |
| Contraceptives tubal ligation | 1 (0.4) | 5 (1.0) | 0.42 (0.05-3.61, 0.41) |
| Vaginal practices | 93 (37.3) | 168 (32.0) | 1.27 (0.92-1.74, 0.14) |
| Parity  0  1-2  3 or more | 52 (20.9)  147 (59.0)  50 (20.1) | 137 (26.2)  276 (52.8)  110 (21.0) | ref*  0.71 (0.49-1.04, 0.08)  0.84 (0.53-1.33, 0.44) |
| Sexual behaviour |  |  |  |
| Condom use last 3 months | 73 (29.3) | 123 (23.4) | 1.36 (0.97-1.90, 0.08) |
| Condom use during last sexual contact | 52 (20.9) | 98 (18.7) | 1.15 (0.79-1.68, 0.47) |
| Number of sexual partners previous 3 months  0 partners  1 partner  2 or more partners | 21 (8.4)  214 (85.9)  14 (5.6) | 58 (11.0)  450 (85.7)  17 (3.2) | ref*  0.76 (0.45-1.29, 0.31)  0.44 (0.19-1.05, 0.06) |
| Number of sexual partners previous 12 months  0 partners  1 partner  2 or more partners | 10 (4.0)  186 (74.7)  53 (21.3) | 23 (4.4)  428 (81.5)  74 (14.1) | ref*  1.00 (0.47-2.14, 1.00)  0.61 (0.27-1.38, 0.23) |
| Days since last sexual contact  0 to 7 days  8 to 14 days  More than 14 days | 112 (47.5)  46 (19.5)  78 (33.1) | 228 (46.1)  74 (14.9)  193 (39.0) | ref*  0.79 (0.51-1.22, 0.29)  1.22 (0.86-1.72, 0.27) |
| Allergies | 21 (8.4) | 32 (6.1) | 1.42 (0.80-2.52, 0.23) |
| BMI  Under/normal weight  Overweight  Obesity | 122 (52.6)  79 (34.1)  31 (13.4) | 243 (49.8)  163 (33.4)  82 (16.8) | ref*  1.04 (0.73-1.46, 0.84)  1.33 (0.83-2.12, 0.23) |
| Medication use previous 4 weeks |  |  |  |
| Antibiotics | 54 (21.7) | 128 (24.4) | 0.86 (0.60-1.23, 0.41) |
| Antifungals | 22 (8.8) | 54 (10.3) | 0.85 (0.50-1.42, 0.53) |
| Steroids | 8 (3.2) | 18 (3.4) | 0.94 (0.40-2.18 (0.88) |
| None | 144 (57.8) | 309 (58.9) | 0.96 (0.71-1.30, 0.79) |

**Legend:** *The OR is calculated for the symptom category ‘yes’ compared to ‘no’, unless stated otherwise. E.g. in the case of a symptom with more than two categories, then the first category was used as a reference.

**Table S4:** Socio-demographic and clinical characteristics in Trichomonas vaginalis (TV) positive and negative cases

|  | TV positive n (%)  n = 76 | No TV n (%)  n = 698 | OR (95% CI, p-value) |
| --- | --- | --- | --- |
| Demographics | | | |
| Age  18 to 25 years  26 to 35 years  36 and above | 22 (29.3)  32 (42.7)  21 (28.0) | 268 (39.2)  276 (40.4)  139 (20.4) | ref*  0.71 (0.40-1.25, 0.23)  0.54 (0.29-1.02, 0.06) |
| Educational level  None/primary  Secondary  Tertiary | 30 (39.5)  31 (40.8)  15 (19.7) | 174 (25.0)  318 (45.6)  205 (29.4) | ref*  1.77 (1.04-3.02, **0.04**)  2.36 (1.23-4.52, **0.01**) |
| Marital status  Single  Married  Separated, divorced or widowed | 20 (26.3)  39 (51.3)  17 (22.4) | 234 (33.6)  385 (55.2)  78 (11.2) | ref*  0.84 (0.48-1.48, 0.55)  0.39 (0.20-0.79, **0.01**) |
| Occupation  Unemployed, housewife  Professional worker  Self employed  Student  Other | 30 (39.5)  6 (7.9)  20 (26.3)  4 (5.3)  16 (21.1) | 239 (34.3)  87 (12.5)  227 (32.6)  56 (8.0)  88 (12.6) | ref* |
| Ethnicity  Other  Luhya  Kikuyu  Kamba | 20 (26.7)  27 (36.0)  23 (30.7)  5 (6.7) | 169 (24.4)  136 (19.6)  280 (40.4)  108 (15.6) | ref*  0.60 (0.32-1.11, 0.10)  1.44 (0.77-2.70, 0.25)  2.56 (0.93-7.01, 0.06) |
| Symptoms |  |  |  |
| Discharge curdy/curdled | 65 (85.5) | 545 (79.0) | 1.57 (0.81-3.06, 0.18) |
| Discharge foul smell | 39 (51.3) | 181 (25.9) | 3.01 (1.86-4.87, **0.00**) |
| Vulvar itch or pruritus | 61 (80.3) | 527 (75.5) | 1.32 (0.73-2.38, 0.36) |
| Lower abdominal pain | 15 (19.7) | 206 (29.5) | 0.59 (0.33-1.06, 0.07) |
| Soreness | 19 (25.0) | 200 (28.7) | 0.83 (0.48-1.43, 0.50) |
| Dysuria | 38 (50.0) | 309 (44.3) | 1.26 (0.78-2.02, 0.34) |
| Dyspareunia | 28 (36.8) | 289 (41.4) | 0.83 (0.51-1.35, 0.44) |
| Recurrent LGTS previous 12 months | 34 (44.7) | 397 (56.9) | 0.61 (0.38-0.99, **0.04)** |
| Signs |  |  |  |
|  |  |  |  |
| Abdominal tenderness | 2 (2.6) | 25 (3.6) | 0.73 (0.17-3.13, 0.67) |
| Excoriations, ulcers or skin lesions | 4 (5.3) | 52 (7.5) | 0.68 (0.24-1.95, 0.47) |
| Erythema/redness | 19 (25.0) | 110 (15.9) | 1.76 (1.01-3.08, **0.04**) |
| Vesicles | 5 (6.6) | 9 (1.3) | 5.34 (1.74-16.38, **0.00**) |
| Oedema | 8 (10.5) | 30 (4.3) | 2.60 (1.15-5.89, **0.02**) |
| Growth warts | 1 (1.3) | 18 (2.6) | 0.50 (0.07-3.79. 0.49) |
| Use of contraceptives | 50 (65.8) | 453 (64.9) | 1.04 (0.63-1.71, 0.88) |
| Contraceptives natural/herbal | 6 (7.9) | 42 (6.0) | 1.34 (0.55-3.26, 0.52) |
| Contraceptives hormonal | 27 (35.5) | 227 (32.5) | 1.14 (0.70-1.88, 0.60) |
| Contraceptives IUCD | 10 (13.2) | 83 (11.9) | 1.12 (0.56-2.27, 0.75) |
| Contraceptives tubal ligation | 1 (1.3) | 5 (0.7) | 1.85 (0.21-16.03, 0.57) |
| Vaginal practices | 33 (43.4) | 228 (32.7) | 1.58 (0.98-2.56, 0.06) |
| Parity  0  1-2  3 or more | 10 (13.2)  41 (53.9)  25 (32.9) | 179 (25.7)  382 (54.9)  135 (19.4) | ref*  5.21 (0.26-1.06, 0.07)  0.30 (0.14-0.65, **0.00**) |
| Sexual behaviour |  |  |  |
| Condom use last 3 months | 19 (25.0) | 177 (25.4) | 0.98 (0.57-1.69, 0.95) |
| Condom use during last sexual contact | 14 (18.4) | 136 (19.5) | 0.93 (0.51-1.72, 0.82) |
| Number of sexual partners previous 3 months  0 partners  1 partner  2 or more partners | 10 (13.2)  62 (81.6)  4 (5.3) | 69 (9.9)  602 (86.2)  27 (3.9) | ref*  1.41 (0.69-2.87, 0.35)  0.98 (0.28-3.39, 0.97) |
| Number of sexual partners previous 12 months  0 partners  1 partner  2 or more partners | 6 (7.9)  55 (72.4)  15 (19.7) | 27 (3.9)  559 (80.1)  112 (16.0) | ref*  2.26 (0.89-5.71, 0.08)  1.66 (0.59-4.68, 0.33) |
| Days since last sexual contact  0 to 7 days  8 to 14 days  More than 14 days | 31 (43.1)  17 (23.6)  24 (33.3) | 309 (46.9)  103 (15.6)  247 (37.5) | ref*  0.61 (0.32-1.14, 0.12)  1.03 (0.59-1.81, 0.91) |
| Allergies | 10 (13.2) | 43 (6.2) | 2.31 (1.11-4.80, **0.02**) |
| BMI  Under/normal weight  Overweight  Obesity | 39 (53.4)  23 (31.5)  11 (15.1) | 326 (50.4)  219 (33.8)  102 (15.8) | ref*  1.14 (0.66-1.96, 0.64)  1.11 (0.55-2.25, 0.77) |
| Medication use previous 4 weeks |  |  |  |
| Antibiotics | 16 (21.1) | 166 (23.8) | 0.86 (0.48-1.52, 0.59) |
| Antifungals | 5 (6.6) | 71 (10.2) | 0.62 (0.24-1.59, 0.32) |
| Steroids | 1 (1.3) | 25 (3.6) | 0.36 (0.05-2.69, 0.30) |

**Legend:** *The OR is calculated for the symptom category ‘yes’ compared to ‘no’, unless stated otherwise. e.g. in the case of a symptom with more than two categories, then the first category was used as a reference.

**Table S5:** Socio-demographic and clinical characteristics in Neisseria Gonorrhoea (NG) positive and negative cases

|  | NG positive n (%)  n = 111 | No NG n (%)  n = 663 | OR (95% CI, p-value) |
| --- | --- | --- | --- |
| Demographics | | | |
| Age  18 to 25 years  26 to 35 years  36 and above | 52 (47.7)  38 (34.9)  19 (17.4) | 238 (36.7)  270 (41.6)  141 (21.7) | ref*  1.55 (0.99-2.44, 0.06)  1.62 (0.92-2.85, 0.09) |
| Educational level  None/primary  Secondary  Tertiary | 24 (21.6)  62 (55.9)  25 (22.5) | 180 (27.2)  287 (43.4)  195 (29.5) | ref*  0.62 (0.37-1.02, 0.06)  1.04 (0.57-1.89, 0.90) |
| Marital status  Single  Married  Separated, divorced or widowed | 39 (35.1)  57 (51.4)  15 (13.5) | 215 (32.5)  367 (55.4)  80 (12.1) | ref*  1.17 (0.75-1.82, 0.49)  0.97 (0.51-1.85, 0.92) |
| Occupation  Unemployed, housewife  Professional worker  Self employed  Student  Other | 41 (36.9)  12 (10.8)  27 (24.3)  13 (11.7)  18 (16.2) | 228 (34.4)  81 (12.2)  220 (33.2)  47 (7.1)  86 (13.0) | ref* |
| Ethnicity  Other  Luhya  Kikuyu  Kamba | 29 (26.4)  34 (30.9)  36 (32.7)  11 (10.0) | 160 (24.3)  129 (19.6)  267 (40.6)  102 (15.5) | ref*  0.69 (0.40-1.19, 0.18)  1.34 (0.79-2.28, 0.27)  1.68 (0.80-3.51, 0.16) |
| Symptoms |  |  |  |
| Discharge curdy/curdled | 80 (74.1) | 530 (80.5) | 0.69 (0.43-1.11, 0.12) |
| Discharge foul smell | 30 (27.0) | 190 (28.7) | 0.92 (0.59-1.45, 0.72) |
| Vulvar itch or pruritus | 83 (74.8) | 505 (76.2) | 0.93 (0.58-1.48, 0.75) |
| Lower abdominal pain | 29 (26.1) | 192 (29.0) | 0.87 (0.55-1.37, 0.54) |
| Soreness | 35 (31.5) | 184 (27.8) | 1.20 (0.78-1.85, 0.41) |
| Dysuria | 55 (49.5) | 292 (44.0) | 1.25 (0.83-1.87, 0.28) |
| Dyspareunia | 47 (42.3) | 270 (40.7) | 1.07 (0.71-1.61, 0.75) |
| Recurrent LGTS previous 12 months | 46 (41.4) | 385 (58.1) | 0.51 (0.34-0.76, **0.00)** |
| Signs |  |  |  |
|  |  |  |  |
| Abdominal tenderness | 5 (4.5) | 22 (3.3) | 1.37 (0.51-3.71, 0.53) |
| Excoriations, ulcers or skin lesions | 4 (4.6) | 51 (7.7) | 0.58 (0.23-1.49, 0.25) |
| Erythema/redness | 14 (13.0) | 115 (17.4) | 0.71 (0.39-1.28, 0.25) |
| Vesicles | 1 (0.9) | 13 (2.0) | 0.47 (0.06-3.59, 0.45) |
| Oedema | 4 (3.7) | 34 (5.2) | 0.71 (0.25-2.04, 0.52) |
| Growth warts | 1 (0.9) | 18 (2.7) | 0.33 (0.04-2.52, 0.26) |
| Use of contraceptives | 70 (63.1) | 433 (65.3) | 0.91 (0.60-1.38, 0.65) |
| Contraceptives natural/herbal | 8 (7.2) | 40 (6.0) | 1.21 (0.55-2.66, 0.64) |
| Contraceptives hormonal | 37 (33.3) | 217 (32.7) | 1.03 (0.67-1.58. 0.90) |
| Contraceptives IUCD | 9 (8.1) | 84 (12.7) | 0.61 (0.30-1.25, 0.17) |
| Contraceptives tubal ligation | 0 (0.0) | 6 (0.9) | 0.86 (0.83-0.88, 0.31) |
| Vaginal practices | 44 (39.6) | 217 (32.7) | 1.35 (0.89-2.04, 0.15) |
| Parity  0  1-2  3 or more | 25 (22.5)  67 (60.4)  19 (17.1) | 164 (24.8)  356 (53.9)  141 (21.3) | ref*  0.81 (0.49-1.33, 0.40)  1.13 (0.60-2.14, 0.70) |
| Sexual behaviour |  |  |  |
| Condom use last 3 months | 35 (31.5) | 161 (24.3) | 1.44 (0.93-2.23, 0.10) |
| Condom use during last sexual contact | 30 (27.0) | 120 (18.1) | 1.68 (1.06-2.66, **0.03**) |
| Number of sexual partners previous 3 months  0 partners  1 partner  2 or more partners | 10 (9.0)  97 (87.4)  4 (3.6) | 69 (10.4)  567 (85.5)  27 (4.1) | ref*  0.85 (0.42-1.70, 0.64)  0.98 (0.28-3.39, 0.97) |
| Number of sexual partners previous 12 months  0 partners  1 partner  2 or more partners | 6 (5.4)  82 (73.9)  23 (20.7) | 27 (4.1)  532 (80.2)  104 (15.7) | ref*  1.44 (0.58-3.60, 0.43)  1.01 (0.37-2.71, 0.99) |
| Days since last sexual contact  0 to 7 days  8 to 14 days  More than 14 days | 47 (45.6)  20 (19.4)  36 (35.0) | 293 (46.7)  100 (15.9)  235 (37.4) | ref*  0.80 (0.45-1.42, 0.45)  1.05 (0.66-1.67, 0.85) |
| Allergies | 8 (7.2) | 45 (6.8) | 1.07 (0.49-2.33, 0.87) |
| BMI  Under/normal weight  Overweight  Obesity | 53 (52.5)  34 (33.7)  14 (13.9) | 312 (50.4)  208 (33.6)  99 (16.0) | ref*  1.04 (0.65-1.65, 0.87)  1.20 (0.64-2.26, 0.57) |
| Medication use previous 4 weeks |  |  |  |
| Antibiotics | 27 (24.3) | 155 (23.4) | 1.05 (0.66-1.69, 0.83) |
| Antifungals | 12 (10.8) | 64 (9.7) | 1.13 (0.59-2.18, 0.70) |
| Steroids | 2 (1.8) | 24 (3.6) | 0.49 (0.11-2.10, 0.33) |

**Legend:** *The OR is calculated for the symptom category ‘yes’ compared to ‘no’, unless stated otherwise. e.g. in the case of a symptom with more than two categories, then the first category was used as a reference.

**Table S6:** Socio-demographic and clinical characteristics in Chlamydia trachomatis (CT) positive and negative cases

|  | CT positive N (%)  n = 97 | No CT N (%)  n = 677 | OR (95% CI, p-value) |
| --- | --- | --- | --- |
| Demographics | | | |
| Age  18 to 25 years  26 to 35 years  36 and above | 60 (62.5)  23 (24.0)  13 (13.5) | 230 (34.7)  285 (43.1)  147 (22.2) | ref*  3.23 (1.94-5.39, **0.00**)  2.95 (1.56-5.56, **0.00**) |
| Educational level  None/primary  Secondary  Tertiary | 21 (21.6)  48 (49.5)  28 (28.9) | 183 (27.1)  301 (44.5)  192 (28.4) | ref*  0.72 (0.42-1.24, 0.26)  0.79 (0.43-1.44, 0.43) |
| Marital status  Single  Married  Separated, divorced or widowed | 40 (41.2)  49 (50.5)  8 (8.2) | 214 (31.7)  375 (55.5)  87 (12.9) | ref*  1.43 (0.91-2.24, 0.12)  2.03 (0.91-4.52, 0.08) |
| Occupation  Unemployed, housewife  Professional worker  Self employed  Student  Other | 36 (37.1)  15 (15.5)  16 (16.5)  15 (15.5)  15 (15.5) | 233 (34.5)  78 (11.5)  231 (34.1)  45 (6.7)  89 (13.2) | ref* |
| Ethnicity  Other  Luhya  Kikuyu  Kamba | 18 (18.8)  32 (33.3)  36 (37.5)  10 (10.4) | 171 (25.4)  131 (19.5)  267 (39.7)  103 (15.3) | ref*  0.43 (0.23-0.80, **0.01**)  0.78 (0.43-1.42, 0.42)  1.08 (0.48-2.44, 0.85) |
| Symptoms |  |  |  |
| Discharge curdy/curdled | 75 (77.3) | 535 (80.0) | 0.85 (0.51-1.42, 0.55) |
| Discharge foul smell | 31 (32.0) | 189 (27.9) | 1.21 (0.77-1.92, 0.41) |
| Vulvar itch or pruritus | 73 (75.3) | 515 (76.1) | 0.96 (0.58-1.57, 0.86) |
| Lower abdominal pain | 24 (24.7) | 197 (29.1) | 0.80 (0.49-1.31, 0.37) |
| Soreness | 31 (32.0) | 188 (27.8) | 1.22 (0.77-1.93, 0.39) |
| Dysuria | 49 (50.5) | 298 (44.0) | 1.30 (0.85-1.99, 0.23) |
| Dyspareunia | 38 (39.2) | 279 (41.2) | 0.92 (0.59-1.42, 0.70) |
| Recurrent LGTS previous 12 months | 44 (45.4) | 387 (57.2) | 0.62 (0.40-0.95, **0.03**) |
| Signs |  |  |  |
|  |  |  |  |
| Abdominal tenderness | 3 (3.1) | 24 (3.5) | 0.87 (0.26-2.94, 0.82) |
| Excoriations, ulcers or skin lesions | 5 (5.2) | 51 (7.6) | 0.66 (0.26-1.70, 0.39) |
| Erythema redness | 12 (12.4) | 117 (17.4) | 0.67 (0.35-1.26, 0.21) |
| Vesicles | 1 (1.0) | 13 (1.9) | 0.53 (0.07-4.08, 0.53) |
| Oedema | 3 (3.1) | 35 (5.2) | 0.58 (0.18-1.92, 0.37) |
| Growth warts | 3 (3.1) | 16 (2.4) | 1.31 (0.37-4.57, 0.68) |
| Use of contraceptives | 65 (67.0) | 438 (64.7) | 1.11 (0.71-1.74, 0.66) |
| Contraceptives natural/herbal | 4 (4.1) | 44 (6.5) | 0.62 (0.22-1.76, 0.36) |
| Contraceptives hormonal | 35 (36.1) | 219 (32.3) | 1.18 (0.76-1.84, 0.46) |
| Contraceptives IUCD | 9 (9.3) | 84 (12.4) | 0.72 (0.35-1.49, 0.38) |
| Contraceptives tubal ligation | 0 (0.0) | 6 (0.9) | 0.87 (0.85-0.90, 0.35) |
| Vaginal practices | 30 (30.9) | 231 (34.1) | 0.87 (0.55-1.37, 0.53) |
| Parity  0  1-2  3 or more | 26 (26.8)  58 (59.8)  13 (13.4) | 163 (24.1)  365 (54.1)  147 (21.8) | ref*  1.00 (0.61-1.65, 0.99)  1.80 (0.89-3.64, 0.10) |
| Sexual behaviour |  |  |  |
| Condom use last 3 months | 30 (30.9) | 166 (24.5) | 1.38 (0.87-2.19, 0.18) |
| Condom use during last sexual contact | 19 (19.6) | 131 (19.4) | 1.02 (0.59-1.74, 0.96) |
| Number of sexual partners previous 3 months  0 partners  1 partner  2 or more partners | 7 (7.2)  83 (85.6)  7 (7.2) | 72 (10.6)  581 (85.8)  24 (3.5) | ref*  0.68 (0.30-1.53, 0.35)  0.33 (0.11-1.05-0.05) |
| Number of sexual partners previous 12 months  0 partners  1 partner  2 or more partners | 1 (1.0)  69 (71.1)  27 (27.8) | 32 (4.7)  545 (80.5)  100 (14.8) | ref*  0.25 (0.03-1.84, 0.14)  0.12 (0.02-0.89 (**0.01**) |
| Days since last sexual contact  0 to 7 days  8 to 14 days  More than 14 days | 40 (42.6)  20 (21.3)  34 (36.2) | 300 (47.1)  100 (15.7)  237 (37.2) | ref*  0.67 (0.37-1.19, 0.17)  0.93 (0.57-1.51, 0.77) |
| Allergies | 9 (9.3) | 44 (6.5) | 1.47 (0.69-3.12, 0.31) |
| BMI  Under/normal weight  Overweight  Obesity | 51 (56.0)  31 (34.1)  9 (9.9) | 314 (49.9)  211 (33.5)  104 (16.5) | ref*  1.11 (0.69-1.79, 0.68)  1.88 (0.89-3.94, 0.09) |
| Medication use previous 4 weeks |  |  |  |
| Antibiotics | 14 (14.4) | 168 (24.8) | 0.51 (0.28-0.92, **0.02**) |
| Antifungals | 8 (8.2) | 68 (10.0) | 0.81 (0.37-1.73, 0.58) |
| None | 63 (64.9) | 390 (57.6) | 1.36 (0.88-2.13, 0.17) |

**Legend:** *The OR is calculated for the symptom category ‘yes’ compared to ‘no’, unless stated otherwise. e.g. in the case of a symptom with more than two categories, then the first category was used as a reference.

**Table S7:** Socio-demographic and clinical characteristics in Mycoplasma genitalium (MG) positive and negative cases

|  | MG positive N (%)  n = 43 | No MG N (%)  n = 731 | OR (95% CI, p-value) |
| --- | --- | --- | --- |
| Demographics | | | |
| Age  18 to 25 years  26 to 35 years  36 and above | 26 (60.5)  11 (25.6)  6 (14.0) | 264 (36.9)  297 (41.5)  154 (21.5) | ref*  2.66 (1.29-5.49, **0.01**)  2.53 (1.02-6.28, **0.04**) |
| Educational level  None/primary  Secondary  Tertiary | 11 (25.6)  15 (34.9)  17 (39.5) | 193 (26.4)  334 (45.8)  203 (27.8) | ref*  1.27 (0.57-2.82, 0.56)  0.68 (0.31-1.49, 0.33) |
| Marital status  Single  Married  Separated, divorced or widowed | 19 (44.2)  22 (51.2)  2 (4.7) | 235 (32.2)  402 (55.1)  93 (12.7) | ref*  1.48 (0.78-2.79, 0.23)  3.76 (0.86-16.5, 0.06) |
| Occupation  Unemployed, housewife  Professional worker  Self employed  Student  Other | 14 (32.6)  8 (18.6)  9 (20.9)  8 (18.6)  4 (9.3) | 255 (34.9)  85 (11.6)  238 (32.6)  52 (7.2)  100 (13.7) | ref* |
| Ethnicity  Other  Luhya  Kikuyu  Kamba | 13 (31.0)  11 (26.2)  10 (23.8)  8 (19.0) | 176 (24.2)  152 (20.9)  293 (40.4)  105 (14.5) | ref*  1.02 (0.44-2.35, 0.96)  2.16 (0.93-5.04, 0.07)  0.97 (0.39-2.42, 0.95) |
| Symptoms |  |  |  |
| Discharge curdy/curdled | 31 (72.1) | 579 (80.1) | 0.64 (0.32-1.28, 0.21) |
| Discharge foul smell | 10 (23.3) | 210 (28.7) | 0.75 (0.36-1.55, 0.44) |
| Vulvar itch or pruritus | 34 (79.1) | 554 (75.8) | 1.21 (0.57-2.57, 0.62) |
| Lower abdominal pain | 11 (25.6) | 210 (28.7) | 0.85 (0.42-1.72, 0.66) |
| Soreness | 11 (25.6) | 208 (28.5) | 0.86 (0.43-1.75, 0.68) |
| Dysuria | 27 (62.8) | 320 (43.8) | 2.17 (1.15-4.09, **0.02**) |
| Dyspareunia | 21 (48.8) | 296 (40.5) | 1.40 (0.76-2.60, 0.28) |
| Recurrent LGTS previous 12 months | 27 (62.8) | 404 (55.3) | 1.37 (0.72-2.63, 0.33) |
| Signs |  |  |  |
|  |  |  |  |
| Abdominal tenderness | 1 (2.3) | 26 (3.6) | 0.65 (0.09-4.87, 0.67) |
| Excoriations, ulcers or skin lesions | 3 (7.0) | 53 (7.3) | 0.95 (0.29-3.18, 0.94) |
| Erythema redness | 8 (18.6) | 121 (16.7) | 1.14 (0.52-2.52, 0.74) |
| Vesicles | 1 (2.3) | 13 (1.8) | 1.30 (0.17-10.21, 0.80) |
| Oedema | 2 (4.7) | 36 (5.0) | 0.93 (0.22-4.01, 0.93) |
| Growth warts | 0 (0.0) | 19 (2.6) | 0.94 (0.93-0.96, 0.28) |
| Use of contraceptives | 31 (72.1) | 472 (64.6) | 1.42 (0.72-2.81), 0.32) |
| Contraceptives natural/herbal | 3 (7.0) | 45 (6.2) | 1.14 (0.34-3.84, 0.83) |
| Contraceptives hormonal | 15 (34.9) | 239 (32.7) | 1.10 (0.58-2.10, 0.77) |
| Contraceptives IUCD | 2 (4.7) | 91 (12.4) | 0.34 (0.08-1.44, 0.13) |
| Contraceptives tubal ligation | 0 (0.0) | 6 (0.8) | 0.94 (0.93-0.96, 0.55) |
| Vaginal practices | 20 (46.5) | 241 (33.0) | 1.77 (0.95-3.28, 0.07) |
| Parity  0  1-2  3 or more | 12 (27.9)  27 (62.8)  4 (9.3) | 177 (24.3)  396 (54.3)  156 (21.4) | ref*  0.99 (0.49-2.01, 0.99)  2.64 (0.84-8.37, 0.09) |
| Sexual behaviour |  |  |  |
| Condom use last 3 months | 19 (44.2) | 177 (24.2) | 0.40 (0.22-0.75, **0.00**) |
| Condom use during last sexual contact | 10 (23.3) | 140 (19.2) | 1.28 (0.62-2.66, 0.51) |
| Number of sexual partners previous 3 months  0 partners  1 partner  2 or more partners | 2 (4.7)  39 (90.7)  2 (4.7) | 77 (10.5)  625 (85.5)  29 (4.0) | ref*  0.42 (0.10-1.76, 0.22)  0.38 (0.05-2.80, 0.32) |
| Number of sexual partners previous 12 months  0 partners  1 partner  2 or more partners | 0 (0.0)  31 (72.1)  12 (27.9) | 33 (4.5)  583 (79.8)  115 (15.7) | ref*  1.05 (1.03-1.07, 0.19)  1.10 (1.04-1.17, 0.07) |
| Days since last sexual contact  0 to 7 days  8 to 14 days  More than 14 days | 19 (46.3)  6 (14.6)  16 (39.0) | 321 (46.5)  114 (16.5)  255 (37.0) | ref*  1.13 (0.44-2.89, 0.81)  0.94 (0.48-1.87, 0.87) |
| Allergies | 3 (7.0) | 50 (6.8) | 1.02 (0.31-3.42, 0.97) |
| BMI  Under/normal weight  Overweight  Obesity | 22 (53.7)  10 (24.4)  9 (22.0) | 343 (50.5)  232 (34.3)  104 (15.3) | ref*  1.49 (0.69-3.20, 0.31)  0.74 (0.33-1.66, 0.47) |
| Medication use previous 4 weeks |  |  |  |
| Antibiotics | 9 (20.9) | 173 (23.7) | 0.85 (0.40-1.82, 0.68) |
| Antifungals | 5 (11.6) | 71 (9.7) | 1.22 (0.47-3.21, 0.68) |
| Steroids | 3 (7.0) | 23 (3.1) | 2.31 (0.67-8.02, 0.18) |

**Legend:** *The OR is calculated for the symptom category ‘yes’ compared to ‘no’, unless stated otherwise. e.g. in the case of a symptom with more than two categories, then the first category was used as a reference.

**Table S8:** Prevalence of symptoms for no infection and any infection cases

|  | No infection N (%)  n = 273 | Infection N (%)  n = 540 | OR (95% CI, p-value) |
| --- | --- | --- | --- |
| Demographics | | | |
| Age  18 to 25 years  26 to 35 years  36 and above | 92 (34.5)  114 (42.7)  61 (22.8) | 214 (40.4)  211 (39.8)  105 (19.8) | ref*  0.80 (0.57-1.11, 0.18)  0.74 (0.50-1.10, 0.14) |
| Educational level  None/primary  Secondary  Tertiary | 75 (27.6)  118 (43.4)  79 (29.0) | 138 (25.6)  245 (45.4)  157 (29.1) | ref*  1.13 (0.79-1.61, 0.51)  1.08 (0.73-1.60, 0.70) |
| Marital status  Single  Married  Separated, divorced or widowed | 85 (31.3)  155 (57.0)  32 (11.8) | 186 (34.4)  290 (53.7)  64 (11.9) | ref*  0.86 (0.62-1.18, 0.34)  0.91 (0.56-1.50, 0.72) |
| Occupation  Unemployed, housewife  Professional worker  Self employed  Student  Other | 96 (35.3)  25 (9.2)  101 (37.1)  15 (5.5)  35 (12.9) | 183 (33.9)  76 (14.1)  159 (29.4)  49 (9.1)  73 (13.5) | ref* |
| Ethnicity  Other  Luhya  Kikuyu  Kamba | 76 (27.9)  43 (15.8)  104 (38.2)  49 (18.0) | 122 (22.8)  124 (23.2)  217 (40.6)  72 (13.5) | ref*  1.80 (1.15-2.82, **0.01**)  1.30 (0.90-1.88, 0.16)  0.92 (0.58-1.45, 0.71) |
| Symptoms |  |  |  |
| Discharge curdy/curdled | 207 (77.2) | 433 (80.8) | 0.81 (0.57-1.15, 0.24) |
| Discharge foul smell | 56 (20.5) | 174 (32.2) | 0.54 (0.39-0.77, **0.00**) |
| Vulvar itch or pruritus | 194 (71.1) | 419 (77.6) | 0.71 (0.51-0.99, **0.04**) |
| Lower abdominal pain | 91 (33.3) | 142 (26.3) | 1.40 (1.02-1.92, **0.04)** |
| Soreness | 62 (22.7) | 170 (31.5) | 0.64 (0.46-0.90, **0.01**) |
| Dysuria | 132 (48.4) | 231 (42.8) | 1.25 (0.94-1.68, 0.13) |
| Dyspareunia | 109 (39.9) | 224 (41.5) | 0.94 (0.70-1.26, 0.67) |
| Recurrent LGTS previous 12 months | 182 (66.7) | 276 (51.1) | 1.92 (1.41-2.56, **0.00)** |
| Signs |  |  |  |
|  |  |  |  |
| Abdominal tenderness | 10 (3.7) | 19 (3.5) | 1.04 (0.48-2.27, 0.92) |
| Excoriations, ulcers or skin lesions | 18 (6.7) | 40 (7.4) | 0.89 (0.50-1.59, 0.70) |
| Erythema redness | 43 (16.0) | 92 (17.1) | 0.92 (0.62-1.37, 0.68) |
| Vesicles | 3 (1.1) | 12 (2.2) | 0.49 (0.14-1.76, 0.27) |
| Oedema | 13 (4.8) | 25 (4.7) | 1.04 (0.52-2.07, 0.91) |
| Growth warts | 5 (1.9) | 15 (2.8) | 0.66 (0.24-1.83, 0.42) |
| Use of contraceptives | 180 (65.9) | 348 (64.4) | 1.07 (0.79-1.45, 0.67) |
| Contraceptives natural/herbal | 16 (5.9) | 34 (6.3) | 0.93 (0.50-1.71, 0.81) |
| Contraceptives hormonal | 90 (33.0) | 177 (32.8) | 1.01 (0.74-1.38, 0.96) |
| Contraceptives IUCD | 28 (10.3) | 68 (12.6) | 0.79 (0.50-1.27, 0.33) |
| Contraceptives tubal ligation | 2 (0.7) | 4 (0.7) | 0.99 (0.18-5.43, 0.99) |
| Vaginal practices | 79 (28.9) | 190 (35.2) | 0.75 (0.55-1.03, 0.07) |
| Parity  0  1-2  3 or more | 69 (25.4)  151 (55.5)  52 (19.1) | 134 (24.9)  290 (53.9)  114 (21.2) | ref*  0.99 (0.70-1.40, 0.95)  1.13 (0.73-1.75, 0.59) |
| Sexual behaviour |  |  |  |
| Condom use last 3 months | 74 (27.1) | 14 (24.8) | 1.13 (0.81-1.57, 0.48) |
| Condom use during last sexual contact | 56 (20.5) | 101 (18.7) | 1.12 (0.78-1.62, 0.54) |
| Number of sexual partners previous 3 months  0 partners  1 partner  2 or more partners | 32 (11.7)  230 (84.2)  11 (4.0) | 50 (9.3)  464 (85.9)  26 (4.8) | ref*  1.29 (0.81-2.07, 0.29)  1.51 (0.66-3.48, 0.33) |
| Number of sexual partners previous 12 months  0 partners  1 partner  2 or more partners | 19 (7.0)  219 (80.2)  35 (12.8) | 17 (3.1)  421 (78.0)  102 (18.9) | ref*  2.15 (1.10-4.22, **0.02**)  3.26 (1.53-6.96, **0.00**) |
| Days since last sexual contact  0 to 7 days  8 to 14 days  More than 14 days | 117 (45.8)  35 (13.8)  102 (40.3) | 240 (46.5)  94 (18.2)  182 (35.3) | ref*  1.30 (0.83-2.03, 0.25)  0.86 (0.62-1.20, 0.38) |
| Allergies | 18 (6.6) | 36 (6.7) | 0.99 (0.55-1.78, 0.97) |
| BMI  Under/normal weight  Overweight  Obesity | 123 (47.3)  92 (35.4)  45 (17.3) | 264 (52.9)  164 (32.9)  71 (14.2) | ref*  0.83 (0.60-1.16, 0.27)  0.74 (0.48-1.13, 0.16) |
| Medication use previous 4 weeks |  |  |  |
| Antibiotics | 63 (23.1) | 129 (23.9) | 0.96 (0.68-1.35, 0.80) |
| Antifungals | 32 (11.7) | 51 (9.4) | 1.27 (0.80-2.03, 0.31) |
| Steroids | 8 (2.9) | 19 (3.5) | 0.83 (0.36-1.92, 0.66) |

**Legend:** *The OR is calculated for the symptom category ‘yes’ compared to ‘no’, unless stated otherwise. e.g. in the case of a symptom with more than two categories, then the first category was used as a reference.

**Table S9:** Multivariate binary logistic regression (last step) vulvovaginal candidiasis

|  | B | S.E. | Wald | OR (95% CI, p-value) |
| --- | --- | --- | --- | --- |
| Itch or pruritus | 0.79 | 0.23 | 11.71 | 2.20 (1.40-3.46, 0.00) |
| Constant | 0.22 | 0.10 | 4.76 |  |

**Table S10:** Multivariate binary logistic regression (last step) Bacterial vaginosis

|  | B | S.E. | Wald | OR (95% CI, p-value) |
| --- | --- | --- | --- | --- |
| Foul smell | 1.29 | 0.26 | 24.05 | 3.63 (2.17-6.07, 0.00) |
| Itch or pruritus | -0.49 | 0.28 | 2.94 | 0.61 (0.35-1.07, 0.09) |
| Dysuria | -0.77 | 0.28 | 7.62 | 0.46 (0.27-0.80, 0.01) |
| Dyspareunia | -0.78 | 0.30 | 6.90 | 0.46 (0,26-0.82, 0.01) |
| Oedema | 1.25 | 0.79 | 2.51 |  |
| Number of sexual partners previous 12 months* | | | | |
| 0 partners | -2.20 | 1.06 | 4.31 | 0.11 (0.01-0.88, 0.04) |
| 1 partner | -3.09 | 1.08 | 8.26 | 0.05 (0.01-0.37, 0.00) |
| Marital status |  |  |  |  |
| Single | 0.46 | 0.30 | 2.34 | 1.59 (0.88-2.87, 0.13) |
| Married | -0.34 | 0.38 | 0.80 | 0.71 (0.33-1.50, 0.37) |
| Ethnicity |  |  |  |  |
| Other | 0,76 | 1,28 | 0,35 | 2.13 (0.17-26.19, 0.56) |
| Luhya | 0,38 | 1,28 | 0,09 | 1.47 (0.12-18.09, 0.77) |
| Kikuyu | 0,70 | 1,28 | 0,30 | 2.02 (0.17-24.75, 0.58) |
| Kamba | 1,71 | 1,34 | 1,63 | 5.52 (0.40-75.93, 0.20) |
| Use of antifungals | 0.01 | 0.42 | 0.00 | 1.01 (0.44-2.31, 0.99) |
| Constant | 3.14 | 1.68 | 3.49 |  |

*Despite being significant, the number of sexual partners was not used in the alternative algorithm since both 0 and 1 partners are preventive of BV, no clinical difference can be made

**Table S11:** Multivariate binary logistic regression (last step) Any STI

|  | B | S.E. | Wald | OR (95% CI, p-value) |
| --- | --- | --- | --- | --- |
| Foul smell | 0.50 | 0.22 | 4.89 | 1.64 (1.06-2.55, 0.03) |
| Lower abdominal pain | 0.55 | 0.24 | 5.00 | 1.73 (1.07-2.79, 0.03) |
| Excoriations, ulcers or genital skin lesions | 0.91 | 0.47 | 3.88 | 2.50 (1.00-6.23, 0.05) |
| Use of contraceptives | 0.82 | 0.39 | 4.46 | 2.27 (1.06-4.88, 0.03) |
| Condom use last sexual contact | -0.09 | 0.27 | 0.11 | 0.92 (0.54-1.54, 0.74) |
| Age |  |  |  |  |
| 18-25 years | -0.92 | 0.87 | 1.13 | 0.40 (0.07-2.17, 0.29) |
| 26-35 years | -0.16 | 0.86 | 0.03 | 0.86 (0.16-4.60, 0.86) |
| 36 and above | -0.42 | 0.88 | 0.23 | 0.66 (0.12-3.65, 0.63) |
| Educational level |  |  |  |  |
| None/primary | -0.40 | 0.27 | 2.08 | 0.67 (0.39-1.15, 0.15) |
| Secondary | 0.00 | 0.34 | 0.00 | 1.00 (0.51-1.96, 1.00) |
| Occupation |  |  |  |  |
| Unemployed, housewife | -0.19 | 0.36 | 0.27 | 0.83 (0.41-1.69, 0.60) |
| Professional worker | 0.34 | 0.28 | 1.41 | 1.40 (0.80-2.43, 0.23) |
| Self employed | -0.80 | 0.45 | 3.11 | 0.45 (0.19-1.09, 0.08) |
| Student | -0.51 | 0.33 | 2.36 | 0.60 (0.31-1.15, 0.12) |
| Recurrent LGTS episodes previous 12 months | 0.81 | 0.21 | 14.33 | 0.45 (0.29-0.68, **0.00)** |
| Constant | 19.72 | 55837,74 | 0.00 |  |

**Table S12:** Multivariate binary logistic regression (last step) Trichomonas vaginalis

|  | B | S.E. | Wald | OR (95% CI, p-value) |
| --- | --- | --- | --- | --- |
| Foul smell | 0.91 | 0.34 | 7.33 | 2.49 (1.29-4.82, 0.01) |
| Lower abdominal pain | 1.10 | 0.46 | 5.78 | 3.02 (1.23-7.42, 0.02) |
| Vesicles* | -3.92 | 0.88 | 19.75 | 0.02 (0.00-0.11, 0.00) |
| Marital status |  |  |  |  |
| Single | 0.90 | 0.48 | 3.44 | 2.45 (0.95-6.34, 0.06) |
| Married | -0.40 | 0.53 | 0.58 | 0.67 (0.24-1.89, 0.45) |
| Use of antifungals in the previous 4 weeks | -1.32 | 0.82 | 2.56 | 0.27 (0.05-1.35, 0.11) |
| Constant | 57.35 | 37921.27 | 0.00 |  |

*Vesicles was not used in the alternative algorithm since only 15 women in the entire dataset had vesicles, too low number for conclusion on predictive value

**Table S13:** Multivariate binary logistic regression (last step) Chlamydia trachomatis

|  | B | S.E. | Wald | OR (95% CI, p-value) |
| --- | --- | --- | --- | --- |
| Ethnicity |  |  |  |  |
| Other | 2.45 | 1.50 | 2.67 | 11.62 (0.61-220.53, 0.10) |
| Luhya | 1.60 | 1.49 | 1.17 | 4.98 (0.27-91.61, 0.28) |
| Kikuyu | 1.72 | 1.47 | 1.36 | 5.57 (0.31-100.24, 0.24) |
| Kamba | 2.57 | 1.56 | 2.71 | 13.04 (0.62-275.59, 0.10) |
| Use of antibiotics previous 4 weeks | -0.79 | 0.42 | 3.50 | 0.45 (0.20-1.04, 0.06) |
| Recurrent LGTS episodes previous 12 months | 0.81 | 0.30 | 7.14 | 0.44 (0.25-0.81, **0.01)** |
| Constant | 56.59 | 30932.55 | 0.00 |  |

**Table S14:** Multivariate binary logistic regression (last step) Neisseria Gonorrhoea

|  | B | S.E. | Wald | OR (95% CI, p-value) |
| --- | --- | --- | --- | --- |
| Soreness | 0,43 | 0,28 | 2,49 | 1.54 (0.90-2.65, 0.11) |
| Excoriations, ulcers or genital skin lesions | 2,28 | 1,55 | 2,15 | 9.78 (0.46-205.53, 0.14) |
| Condom use | 0,48 | 0,28 | 2,99 | 1.61 (0.94-2.76, 0.08) |
| IUCD use | -0,78 | 0,49 | 2,48 | 0.46 (0.17-1.21, 0.12) |
| Educational level | | | | |
| None or primary | -0,70 | 0,33 | 4,54 | 0.50 (0.26-0.95, 0.03) |
| Secondary | -0,03 | 0,38 | 0,01 | 0.97 (0.46-2.07, 0.94) |
| Recurrent LGTS episodes previous 12 months | 0,78 | 0,26 | 9,03 | 0.46 (0.27-0.76, **0.00)** |
| Constant | -0,91 | 1,66 | 0,30 |  |

**Table S15:** Multivariate logistic regression (last step) Mycoplasma genitalium

|  | B | S.E. | Wald | OR (95% CI, p-value) |
| --- | --- | --- | --- | --- |
| Lower abdominal pain | -0.20 | 0.47 | 0.18 | 0.82 (0.33-2.05, 0.67) |
| Soreness | -0.86 | 0.53 | 2.60 | 0.42 (0.15-1.20, 0.11) |
| Condom use previous 3 months | -0.94 | 0.43 | 4.73 | 0.39 (0.17-0.91, 0.03) |
| Constant | 75.97 | 23302.63 | 0.00 |  |

**Table S16:** Multivariate logistic regression (last step) no infection

|  | B | S.E. | Wald | OR (95% CI, p-value) |
| --- | --- | --- | --- | --- |
| Foul smell | -0.78 | 0.24 | 10.32 | 0.46 (0.29-0.74, 0.00) |
| Itch or pruritus | -0.47 | 0.25 | 3.50 | 0.62 (0.38-1.02, 0.06) |
| Lower abdominal pain | -0.48 | 0.23 | 4.39 | 0.62 (0.39-0.97, 0.04) |
| Vulvar soreness | -0.44 | 0.25 | 3.17 | 0.65 (0.40-1.05, 0.08) |
| Dysuria | 0.37 | 0.22 | 2.96 | 1.45 (0.95-2.22, 0.09) |
| Vesicles | 1.61 | 1.53 | 1.11 | 5.01 (0.25-100.24, 0.29) |
| Condom use | 0.44 | 0.24 | 3.29 | 1.55 (0.97-2.48, 0.07) |
| Number of sexual partners previous 12 months* |  |  |  |  |
| 0 partners | 1.01 | 0.44 | 5.24 | 2.73 (1.16-6.46, 0.02) |
| 1 partner | 1.32 | 0.51 | 6.75 | 3.75 (1.38-10.14, 0.01) |
| Educational level |  |  |  |  |
| None or primary | 0.31 | 0.26 | 1.44 | 1.36 (0.82-2.24, 0.23) |
| Secondary | -0.12 | 0.31 | 0.16 | 0.88 (0.48-1.62, 0.69) |
| Occupation |  |  |  |  |
| Unemployed, housewife | 0.48 | 0.38 | 1.57 | 1.62 (0.76-3.42, 0.21) |
| Professional worker | -0.51 | 0.26 | 3.90 | 0.60 (0.36-1.00, 0.05) |
| Self employed | 0.37 | 0.45 | 0.66 | 1.44 (0.60-3.50, 0.42) |
| Student | 0.21 | 0.33 | 0.41 | 1.24 (0.64-2.38, 0.52) |
| Use of antibiotics previous 4 weeks | -0.50 | 0.25 | 3.94 | 0.61 (0.37-0.99, 0.05) |
| Recurrent LGTS episodes previous 12 months | -0.70 | 0.22 | 10.46 | 2.00 (1.32-3.13, 0.00) |
| Constant | 20.69 | 27645.71 | 0.00 |  |

*Despite being significant, the number of sexual partners was not used in the alternative algorithm since both 0 and 1 partners are preventive of BV, no clinical difference can be made

**Table S17:** McNemar test - computation comparing treatment allocation for LGTI/syndrome, by the current and alternative algorithms (n=306)

| **Treatment category** | **Alternative algorithm** | **Current algorithm** | |  | **χ², P-value** |
| --- | --- | --- | --- | --- | --- |
| VVC (n=121) |  | Correct | Incorrect | Total |  |
|  | Correct | 78 | 24 | 102 | 4.11, 0.04 |
|  | Incorrect | 11 | 8 | 19 |  |
|  | Total | 89 | 32 | 121 |  |
| BV-TV (n=66) |  | Correct | Incorrect | Total |  |
|  | Correct | 23 | 8 | 31 | 5.63, 0.02 |
|  | Incorrect | 22 | 13 | 35 |  |
|  | Total | 45 | 21 | 66 |  |
| LAP (64) |  | Correct | Incorrect | Total |  |
|  | Correct | 20 | 0 | 20 | 0.5, 0.5 |
|  | Incorrect | 2 | 42 | 44 |  |
|  | Total | 22 | 42 | 64 |  |
| No treatment (n=108) |  | Correct | Incorrect | Total |  |
|  | Correct | 0 | 15 | 15 | 13.07, p<0.001 |
|  | Incorrect | 0 | 93 | 93 |  |
|  | Total | 0 | 108 | 108 |  |

Correct: those with the condition and classified to treatment for it.

Incorrect: those with the condition but classified as not having the condition hence missed treatment for it.

Inappropriate treatment was – per definition – not included in the analysis

LGTI: Lower genital tract infections

VVC: Vulvovaginal candidiasis

BV-TV: Bacterial vaginosis-Trichomonas vaginalis

LAP: Lower abdominal pain; includes any of Neisseria gonorrhoea, Chlamydia trachomatis, Mycoplasma genitalium
